# A gap-free genome assembly of *Chlamydomonas reinhardtii* and detection of translocations induced by CRISPR-mediated mutagenesis

**DOI:** 10.1016/j.xplc.2022.100493

**Published:** 2022-11-17

**Authors:** Zachary L. Payne, Gervette M. Penny, Tychele N. Turner, Susan K. Dutcher

**Affiliations:** Department of Genetics, Washington University School of Medicine, Saint Louis, MO 63110, USA

**Keywords:** alga, reference genome, *de novo*, PacBio, Nanopore, structural variation

## Abstract

Genomic assemblies of the unicellular green alga *Chlamydomonas reinhardtii* have provided important resources for researchers. However, assembly errors, large gaps, and unplaced scaffolds as well as strain-specific variants currently impede many types of analysis. By combining PacBio HiFi and Oxford Nanopore long-read technologies, we generated a *de novo* genome assembly for strain CC-5816, derived from crosses of strains CC-125 and CC-124. Multiple methods of evaluating genome completeness and base-pair error rate suggest that the final telomere-to-telomere assembly is highly accurate. The CC-5816 assembly enabled previously difficult analyses that include characterization of the 17 centromeres, rDNA arrays on three chromosomes, and 56 insertions of organellar DNA into the nuclear genome. Using Nanopore sequencing, we identified sites of cytosine (CpG) methylation, which are enriched at centromeres. We analyzed CRISPR-Cas9 insertional mutants in the *PF23* gene. Two of the three alleles produced progeny that displayed patterns of meiotic inviability that suggested the presence of a chromosomal aberration. Mapping Nanopore reads from *pf23-2* and *pf23-3* onto the CC-5816 genome showed that these two strains each carry a translocation that was initiated at the *PF23* gene locus on chromosome 11 and joined with chromosomes 5 or 3, respectively. The translocations were verified by demonstrating linkage between loci on the two translocated chromosomes in meiotic progeny. The three *pf23* alleles display the expected short-cilia phenotype, and immunoblotting showed that *pf23-2* lacks the PF23 protein. Our CC-5816 genome assembly will undoubtedly provide an important tool for the *Chlamydomonas* research community.

## Teaser

The ultimate goal of genome sequencing is to connect the genome to phenotypes of interest. This study reports the generation of a telomere-to-telomere assembly of *Chlamydomonas* by using both PacBio HiFi and Nanopore sequencing, that will help with further mechanistic studies of this model organism. In addition, it shows that CRISPR editing generates a high frequency of translocations in *Chlamydomonas*.

## Introduction

The haploid nuclear genome of the unicellular green alga *Chlamydomonas reinhardtii* resides in 17 chromosomes with a total base-pair length of approximately 120 Mb (Merchant et al., 2007). *Chlamydomonas* has been a valuable model organism for the study of conserved cilia functions (Meng and Pan, 2017), photosynthesis (Rochaix, 2001), biofuel production (Scranton et al., 2015), and sexual reproduction (Goodenough et al., 2007). The *Chlamydomonas* research community is currently equipped with a wide range of tools and techniques at their disposal to facilitate the study and manipulation of this organism (Mussgnug, 2015), including a mutant library derived from insertional mutagenesis (Li et al., 2019), molecular toolkits for cloning and synthetic biology (Crozet et al., 2018; Emrich-Mills et al., 2021), targeted gene-editing strategies (Ferenczi et al., 2017; Akella et al., 2021), and an annotated reference genome (Merchant et al., 2007; Blaby et al., 2014).

The US Department of Energy (DOE) Joint Genome Institute (JGI) genomic assembly of *C. reinhardtii* has provided an important resource for *Chlamydomonas* researchers (Merchant et al., 2007; Blaby et al., 2014). Despite over a decade of improvements to the assembly, including the use of BACs and fosmids to anchor unplaced sequences, the current version (https://phytozome-next.jgi.doe.gov/info/Creinhardtii_v5_6 [v5.6]) is contained in 53 scaffolds assembled from >1500 contigs. Nearly 2 Mb of sequence remains unplaced. Multiple regions in the assembly are not representative of wild-type strains CC-124 and CC-125 owing to either mis-assemblies or chromosomal rearrangements specific to strain CC-503, which was used for sequencing. These shortcomings contributed to our decision to use sequencing platforms that produce reads >10 kb in length. Concurrently, a PacBio version of the genome of CC-4532 is available on BioRxiv (Craig et al., 2022) and at Phytozome (https://phytozome-next.jgi.doe.gov/info/CreinhardtiiCC_4532_v6_1). It is contained in 60 scaffolds.

Genomes assembled from short-read technologies, although highly accurate (>99.9%), are often unable to resolve tandemly or segmentally duplicated sequences, low-complexity regions, and other repetitive sequences within genomes when these sequences are longer than the reads themselves (Mahmoud et al., 2019; Wang et al., 2021a). Thus, assemblies obtained from only short reads tend to contain gaps and short or unplaced contigs. Long-read, single-molecule sequencing technologies from Pacific Biosciences (PacBio) and Oxford Nanopore Technologies (ONT) have revolutionized many aspects of genomics, including improvements to reference genome assembly projects (Miga et al., 2020; Logsdon et al., 2021; Neale et al., 2022), structural variant analyses (Cretu Stancu et al., 2017; Sedlazeck et al., 2018; Hiatt et al., 2021; Zhang et al., 2021), and comparative genomics (Craig et al., 2021; Kim et al., 2021). The balance of accuracy and read length that has been achieved by HiFi circular consensus sequencing (CCS) from PacBio and Nanopore sequencing from ONT improves the ability of assembly programs to reconstruct sequence data obtained from these technologies. Read lengths range from 10 to 20 kb with HiFi and reach hundreds of kilobases or even megabases with ONT, depending on the quality of isolated DNA and the library preparation method (Payne et al., 2019; Logsdon et al., 2020). These reads can span many difficult-to-assemble regions and provide insights into previously unassembled portions of genomes from many organisms. Each of these sequencing technologies has its strengths and limitations. For example, Nanopore reads can reach unprecedented read lengths. However, the base-level quality is only ∼95% accurate. On the other hand, although HiFi reads are ∼99% accurate, they are shorter in length than Nanopore reads (Logsdon et al., 2020). Recently, highly contiguous or even telomere-to-telomere genome assemblies have been generated for human as well as a variety of crops and model organisms by combining long-read technologies, including the telomere-to-telomere assembly of the human genome (Miga et al., 2020; Logsdon et al., 2021; Nurk et al., 2022), watermelon (Deng et al., 2022), rye (Li et al., 2021a; 2021b), rice (Li et al., 2021a; 2021b; Song et al., 2021), *Arabidopsis thaliana* (Wang et al., 2021b; Hou et al., 2022), and multiple drosophilid species (Kim et al., 2021). These assemblies utilized complementary features of more than one technology to assemble highly contiguous and even telomere-to-telomere chromosomes in some cases. Multiple variations of hybrid strategies have been successful. One attractive strategy employed for the assembly of human chromosome 8 combines the use of HiFi and Nanopore long-read technologies (Logsdon et al., 2021). A large fraction of the assembly is composed of contigs derived from HiFi reads. The remaining gaps are composed of sequences derived from Nanopore reads. These were merged to produce a highly accurate and contiguous assembly (Logsdon et al., 2020). The success of hybrid approaches has spurred the development of a plethora of publicly available bioinformatic tools aiming to maximize the strengths of each sequencing technology. Various strategies have been streamlined for labs to include in the assembly work flow (Chakraborty et al., 2016; Koren et al., 2017; Xu et al., 2020). As a result, there has been an influx of new assemblies for unsequenced organisms, as well as improvements to existing assemblies.

We used a combination of high-coverage HiFi and Nanopore data coupled with multiple assembly strategies to develop a reference-quality *C. reinhardtii* genome assembly *de novo* using strain CC-5816, derived from a meiotic cross between wild-type strains CC-124 and CC-125 that was subsequently backcrossed to CC-124 three times. The final assembly consists of 17 scaffolds that correspond to each of the chromosomes and contains no gaps. We have assessed several regions; these include repetitive regions on chromosomes 11 and 15 that are not present in current *Chlamydomonas* assemblies, highly methylated centromeric regions, and insertions of organellar DNA from both the mitochondria (nuclear integrants of mitochondrial DNA [NUMTs]) and chloroplast (nuclear integrants of plastid DNA [NUPTs]) present throughout the genome.

Analysis of structural rearrangements, especially when proximal to repetitive or duplicated sequences, remains difficult with fragmented assemblies. We have taken advantage of this genome assembly as a reference to examine CRISPR-Cas9-generated strains. Meiotic analysis suggests that the genome editing generated large chromosomal aberrations in two of three CRISPR-Cas9-mediated strains from our group and three of six obtained from other groups. We used Nanopore sequencing of the strains generated in our lab to identify the lesions as a translocation between chromosomes 5 and 11 in one strain and between chromosomes 3 and 11 in the other.

## Results

### Assembly of the CC-5816 genome using PacBio HiFi and Nanopore reads

We sought to establish a new *Chlamydomonas* reference genome, given the shortcomings of the current CC-503 v5 assembly (Supplemental Figure 1). The unusually high GC nucleotide content of *Chlamydomonas* (64%) presents a considerable challenge for many sequencing platforms, particularly amplification-based methods (Dohm et al., 2008). The presence of a cell wall creates another challenge with respect to isolating DNA of sufficient quality, quantity, and length required for long-read sequencing. The strain used for the initial genome project, CC-503, was isolated following mutagenesis with the goal of producing mutants with reduced cell wall integrity (Hyams and Davies, 1972), which helped to increase DNA isolation yield (Blaby et al., 2014). However, it is now clear that this method has produced undesired structural changes in the genome (Craig et al., 2022). We reasoned that single-molecule-based methods for sequencing would circumvent issues of nucleotide bias in the final dataset. In addition, we optimized an existing high-molecular-weight (HMW) DNA isolation protocol utilizing the QIAGEN MagAttract HMW DNA Isolation Kit to improve the size and yield of DNA fragments necessary for long-read sequencing technologies without the use of a cell-wall mutant (Supplemental Figure 2).

**Figure 1:**
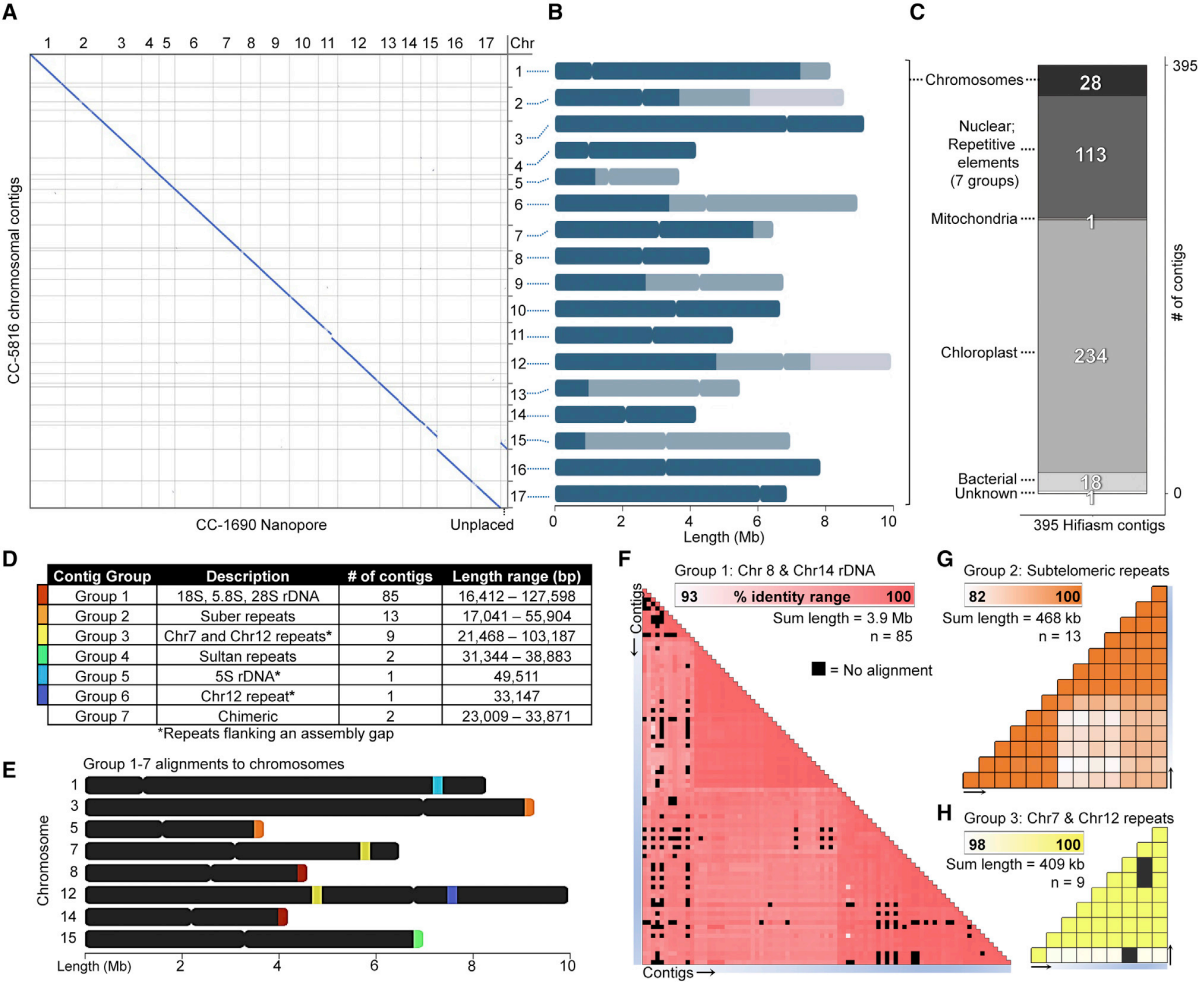
Hifiasm assembly generates a highly contiguous assembly using PacBio HiFi data. **(A)** Dot plot of the 28 largest contigs from CC-5816 that align completely and uniquely to the CC-1690 genome assembly. An unplaced scaffold in the CC-1690 genome assembly was assembled onto chromosome 15 in CC-5816. All alignments were reoriented to the same direction to aid in visualization. **(B)** Ideogram plots of the 28 largest contigs scaffolded into 17 chromosomes. Contigs were scaffolded based on synteny to CC-1690. Contigs are represented as solid colors on each chromosome ranging from dark to light blue. The borders between colors correspond to an assembly gap between contigs. **(C)** Bar chart categorizing the possible origins of all contigs based on alignments to CC-1690, chloroplast and mitochondrial sequences, and potential contaminants. **(D)** Each of the 113 nuclear repetitive contigs were grouped based on the type of repetitive sequence found on each. The two contigs from group 7 had poor read coverage and may derive from low-abundance chimeric reads or transposition events. **(E)** Ideogram plots illustrating approximate locations within the nuclear genome where repetitive contigs from groups 1–6 align. **(F–H)** Percentage identity heatmaps among the three most abundant repetitive contigs filtered from the final assembly are shown. **(F)** rDNA arrays map to one end of both chromosomes 8 and 14, **(G)** Suber repeats appear proximal to telomeric sequence, and **(H)** a repetitive element flanking assembly gaps on chromosomes 7 and 12.

We sequenced *C. reinhardtii* wild-type strain CC-5816 on one PacBio HiFi SMRT cell and obtained ∼18 Gb of CCS reads at approximately 150× coverage of the estimated 120-Mb nuclear genome (Supplemental Figure 3). We used both hifiasm (Cheng et al., 2021) and Canu (Koren et al., 2017; Nurk et al., 2020) assemblers for *de novo* assembly at various coverages (Supplemental Figure 4A). We utilized a Nanopore assembly of strain CC-1690 (also referred to as 21gr) for validation of our assemblies (O’Donnell et al., 2020) (Supplemental Figure 4B). CC-1690 shares a close lineage with CC-5816. It was derived from the meiotic products of a single zygote isolated in 1945 by G.M. Smith in Massachusetts (Pröschold et al., 2005; Harris et al., 2009). Whole-genome alignment revealed that CC-5816 is highly syntenic with CC-1690. A hifiasm assembly using the entire dataset produced the most contiguous assembly and contained no obvious mis-assemblies compared with CC-1690 (Supplemental Figure 4; Supplemental Table 1). We obtained a raw genome assembly of 395 contigs that covered 99.34% of the CC-1690 nuclear sequence by whole-genome alignment (Figure 1A). Nine of the largest contigs make up 50% of the assembly. They range in length from 5 298 489 to 9 193 794 bp. By comparison, the CC-503 v5.6 genome assembly at the contig level contains 1547 contigs, with the 141 longest contigs ranging in length between 215 409 and 1 410 767 bp, making up 50% of the assembly. The 28 longest hifiasm contigs contained 99.28% of the alignments covering CC-1690 nuclear base sequences (Figure 1B) and align to the 17 chromosomes.

We identified 235 contigs containing predominantly organellar sequence and compared them with consensus genomes previously assembled from mitochondria and chloroplasts derived from *Chlamydomonas* (Gallaher et al., 2018). A single mitochondrial (MT) contig in the hifiasm draft assembly was 30 743 bp, twice the length of the consensus ∼15.8-kb linear mitochondrial genome (Gallaher et al., 2018). We determined that this was a mis-assembly from hifiasm, as we did not observe this mis-assembly in the MT contig generated with Canu. We confirmed this mis-assembly by inspecting read coverage across the junction connecting the duplicate MT genomes and observed a lack of reads connecting the two terminal inverted repeats. Alignment of this contig to the consensus MT genome did not reveal any sequence variation. The chloroplast (CP) was represented by 234 contigs ranging in size from ∼21.7 to 93.4 kb. No single contig represented the entire ∼205-kb chloroplast genome, likely because of the abundance of repetitive sequence (Gallaher et al., 2018). One hundred percent of bases from the CP consensus genome aligned to this set of CP contigs, and 99.93% of bases from the contigs produced an alignment. These results confirm the accuracy of the *Chlamydomonas* MT and CP genomes from Gallaher et al. (2018) and suggest few differences among strains. Eighteen contigs ranging in length from 26 083 to 71 538 bp originate from *Chryseobacterium*; these may be contaminants. There is one short, 1533-bp contig of unknown origin (Figure 1C).

**Figure 2:**
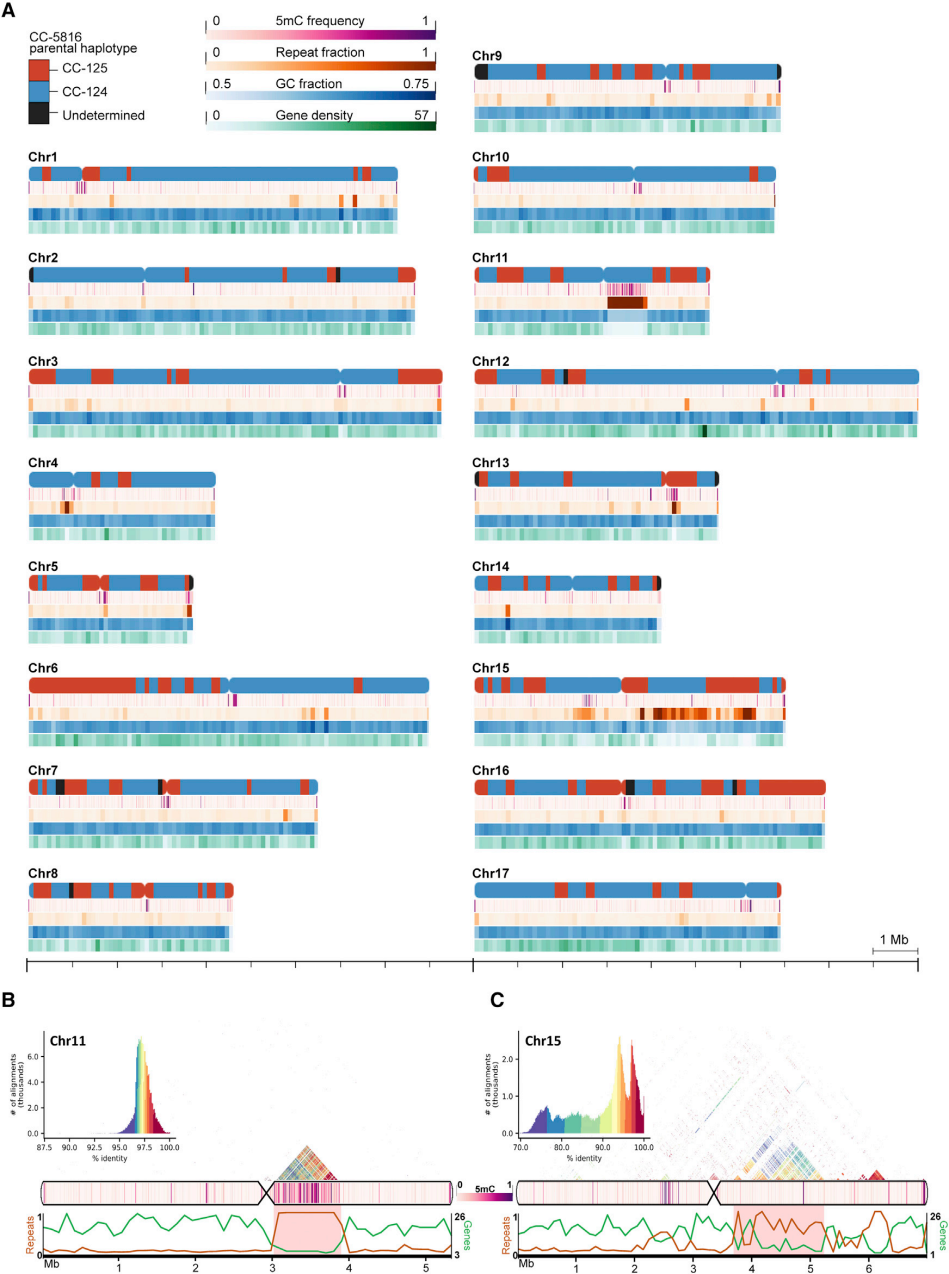
Telomere-to-telomere assembly of CC-5816. **(A)** Gap-free, telomere-to-telomere genome assembly of *C. reinhardtii* mating-type plus strain CC-5816. CC-5816 was obtained from a single meiotic product from multiple crosses between wild-type strains CC-125 and CC-124 and then backcrossed three times to CC-124. Genome features are shown for each of the 17 chromosomes. Approximate centromere locations are based on the location of *ZeppL-1_cRei* clusters and tetrad analysis and are denoted by inward curvature. For each chromosome, five plots from top to bottom show the parental haplotype blocks (red, blue, and black), 5-methylcytosine (5mC) frequency (magenta), repeat fraction determined by Tandem Repeats Finder (orange), GC fraction (blue), and gene density (green) plotted along the lengths, with a scale bar at the bottom for reference. The parental haplotype was determined by creating libraries of 16-mers from the CC-5816 assembly as well as short-read Illumina data from CC-125 and CC-124. 16-mers unique to one of the two parental strains were used to determine phase blocks. Repeat and GC fractions were calculated by the fraction of bases masked with Tandem Repeats Finder and the fraction of G and C nucleotides within 100-kb nonoverlapping windows across each chromosome. Gene density was calculated by counting the number of unique gene loci per 100-kb window. **(B and C)** Ideogram and plots for chromosomes 11 and 15 displaying several features (top to bottom): distribution and heatmap of intrachromosomal sequence identity from all-by-all alignment of 2-kb windows across each chromosome (color scales are independent for each chromosome), 5mC frequency heatmap (magenta), and line plots of repeat fraction (orange) and number of genes (green) per 100-kb window. Highly repetitive regions on each chromosome mentioned in the main text are highlighted in red within the line plots. Note that the appearance of some regions containing larger or inconsistent window sizes compared with others is due to consecutive windows with equivalent values. For example, the repetitive region between 3 and 4 Mb on chromosome 11 appears to have a larger window size in many of the data tracks, but this is due to multiple windows with equivalent values next to each other.

**Figure 3:**
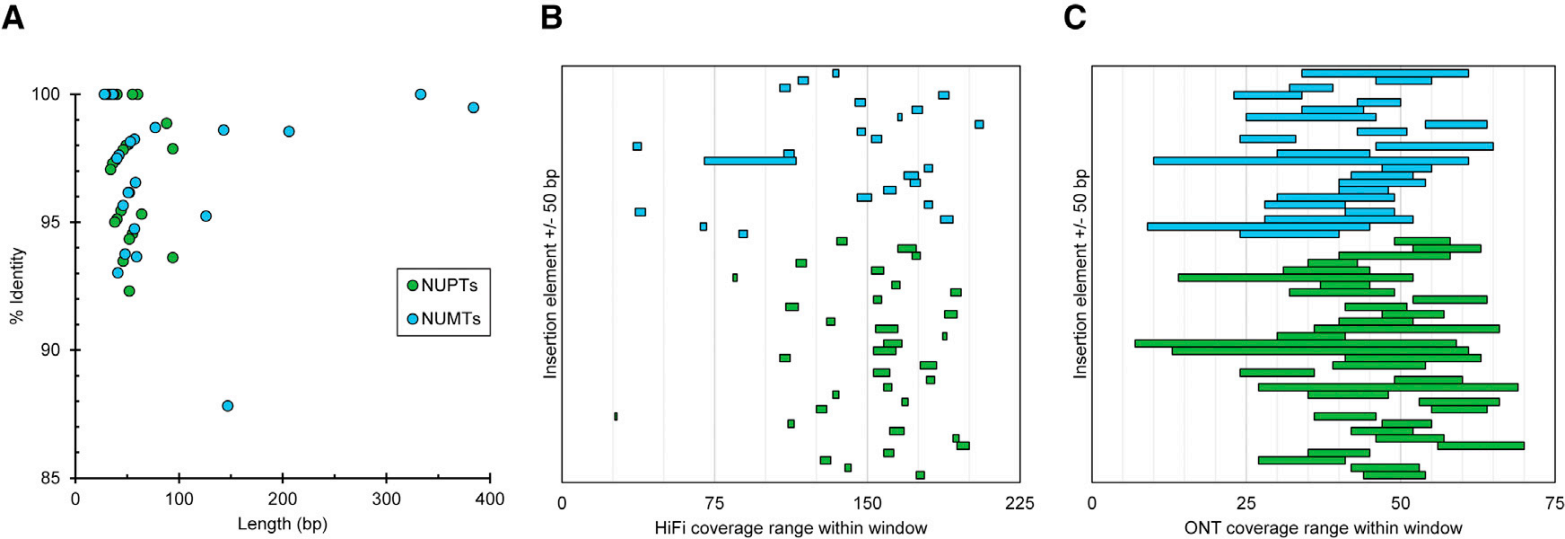
Characterization of organellar sequences in the nuclear genome. **(A)** Organellar insertions plotted by their length (x axis) and percentage identity by BLASTn to their respective *Chlamydomonas* organelle (y axis). **(B)** The range of per-base coverage (x axis) across each organellar insertion ± 50 bp (y axis) from PacBio HiFi reads. **(C)** The range of per-base coverage (x axis) across each organellar insertion ± 50 bp (y axis) from ONT reads. In each panel, NUPTs are colored green and NUMTs are colored blue.

**Figure 4:**
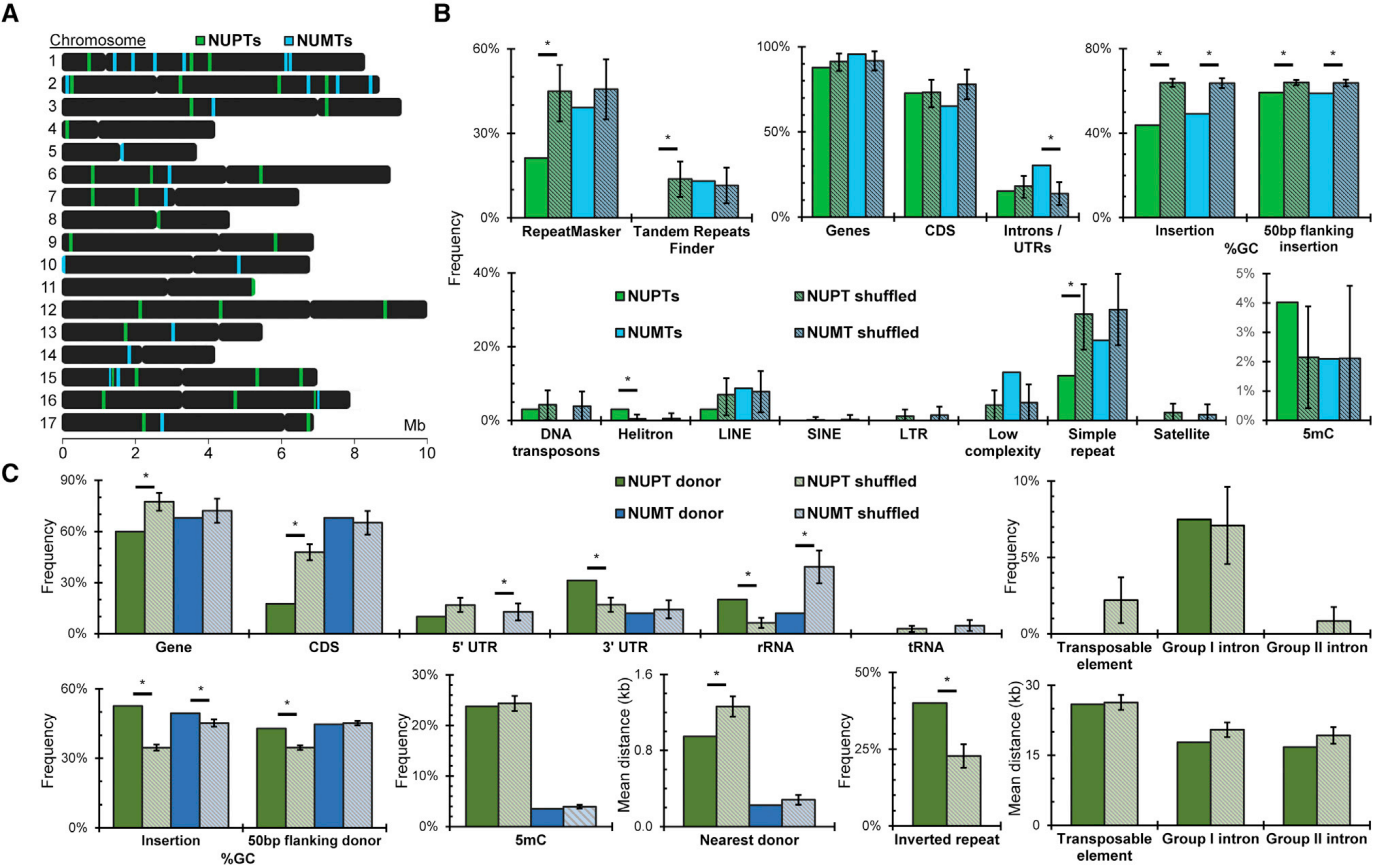
NUPTs and NUMTs originate from specific sequences and are located near specific elements within the nuclear genome. **(A)** Ideogram showing approximate nuclear locations of nuclear integrants of plastid DNA (NUPTs, green) and nuclear integrants of mitochondrial DNA (NUMTs, blue). The widths of colored lines are resized for easy identification and are not correlated with insertion lengths. **(B)** Frequency of genomic elements proximal to NUPTs (green) and NUMTs (blue) within nuclear sequence compared with 100 sets of randomly shuffled coordinates of equal sizes (NUPT simulations, green with black stripes; NUMT simulations, blue with black stripes). **(C)** (Top row, left to right) Frequency of plastid NUPT donors (dark green) and mitochondrial NUMT donors (dark blue) within 50 bp of annotated genes, CDS, 5′ UTRs, 3′ UTRs, rRNAs, and tRNAs. Frequency of NUPT donors within 50 bp of mobile elements, including TEs, group I, and group II introns. (Bottom row, left to right) Frequency of G and C bases within NUPT and NUMT donor sequences and the flanking 50 bp. Per-base frequency of 5mC detected at CpGs within NUPT and NUMT donors. Mean distance between each NUPT and NUMT donor and the nearest donor. Frequency of NUPT donors within 50 bp of either of the two inverted repeats. Mean distance between each NUPT donor and the nearest transposable element, group I, and group II intron. Each is compared with 100 sets of randomly shuffled coordinates of equal sizes (NUPT donor simulations, pale green with black stripes; NUMT simulations, pale blue with black stripes). Plastid annotations were duplicated upstream and downstream for these analyses to account for circular genome conformation. Error bars in **(B and C)** depict standard error of the mean (SEM) for 100 simulated NUPTs and NUMTs. ∗*p* < 0.05 from a two-tailed one-sample *t*-test assuming a normal distribution.

The remaining 113 contigs collectively contain 4 985 725 bp. Each of these contigs shows >99% alignment coverage from the 28 chromosomal contigs using BLAST and 89.53% alignment using NUCmer. These 113 contigs are extremely short; the largest is 127 598 bp (Figure 1D). Multiple sequence alignment of these contigs revealed that 109 of the contigs fall into four groups based on high sequence identity to each other, whereas the four remaining contigs are dissimilar to each other and to the other 109 contigs (Figure 1D). Inspection of repeat content using Tandem Repeats Finder (Benson, 1999) and RepeatMasker (Smit et al., 2013) revealed that this set of 113 contigs contains ∼66% repetitive sequence (compared with ∼22% genome-wide). We used BLAST and NUCmer alignments to determine the genomic origins of these contigs and found that, consistent with the multiple sequence alignment, they fall into seven groups (Figure 1E). They are identified as (1) 18S, 5.8S, 28S ribosomal DNA (rDNA) arrays (Figure 1F), (2) subtelomeric Suber repeats (Figure 1G), (3) repeats flanking two assembly gaps on chromosomes 7 and 12 (Figure 1H), (4) subtelomeric Sultan repeats (Chaux-Jukic et al., 2021), (5) 5S arrays spanning the assembly gap on chromosome 1, (6) repeats flanking a second assembly gap on chromosome 12, and (7) chimeric mis-assemblies or transposition events. We refer to these as groups 1–7, respectively. Group 7 contigs contained transposable elements (TEs) embedded between short nonadjacent nuclear sequences. Only three and five reads mapped to these contigs and were not considered further. The initial draft assembly, consisting of the 28 chromosomal contigs, was evaluated for completeness using BUSCO (Manni et al., 2021) and Augustus (Stanke and Morgenstern, 2005) to identify highly conserved universal single-copy Chlorophyta orthologs (referred to as BUSCOs). We identified 1516 of the 1519 (99.7%) BUSCOs. Addition of the filtered contigs in this evaluation did not recover any additional BUSCOs. The consensus quality value (QV) was evaluated by comparison of k-mers between the 28 contigs and the read set using Merqury (Rhie et al., 2020). This produced a genome-wide QV score of 60.07, or one incorrect base every 1 015 521 bp. These results confirm that a full genomic representation of all unique CC-5816 sequence was indeed present within the 28 largest contigs in the assembly. After scaffolding, only 11 gaps remained between adjacent contigs, which correspond to the 17 chromosomes of *C. reinhardtii* (Figure 1B).

To fill in the remaining 11 gaps in the assembly, we sequenced CC-5816 using Nanopore technology. Six Flongle flow cells were used to optimize our protocol prior to sequencing on an R9 flow cell. In total, we obtained roughly 6.5 Gb of sequence, or approximately 50× coverage, with a read N50 of 39 357 bp and a maximum read size of 338 467 bp (Supplemental Figure 3). We assessed the results of various gap-filling strategies utilizing the Nanopore reads. We assembled the base-called reads using three different assemblers: Canu, Flye (Kolmogorov et al., 2019), and NECAT (Chen et al., 2021a). Eighteen combinations of hybrid Nanopore assemblies were generated using QuickMerge (Chakraborty et al., 2016; Solares et al., 2018). QuickMerge uses sequence from a donor assembly to fill gaps in an acceptor assembly. Using QuickMerge, the HiFi assembly was gap filled with either one of the hybrid ONT assemblies or iteratively using the Canu, Flye, and NECAT assemblies in various combinations to create a total of 20 HiFi–Nanopore hybrid assemblies. We also tested a more conservative gap-filling strategy using TGS-GapCloser (Xu et al., 2020). This method fills gaps using sequence from individual reads that align to the ends of two adjacent contigs. Two assemblies were generated using TGS-GapCloser; the first contained HiFi and Nanopore reads, whereas the second used only Nanopore reads. Finally, to ensure accurate sequence consensus and removal of potential artifacts generated from these gap-filling pipelines, RaCon was used for polishing with aligned HiFi and Nanopore reads in different combinations. All assemblies were carefully assessed for completeness and accuracy at each step by noting the number of gaps filled and checking for recovery or loss of BUSCOs, concordance of k-mers between reads and assembly for QV scores, and improvements in coverage of HiFi and Nanopore reads. Assemblies with significantly reduced metrics in any of the previously listed categories were not considered further. Only assemblies with zero gaps were considered as candidate assemblies for final curation. In total, 43 gapless assemblies were considered for final curation (Supplemental Table 2). An assembly using TGS-GapCloser to fill gaps using first HiFi reads, then Nanopore reads, and no RaCon polishing (assembly titled v0.2 in Supplementary Table 2) had the highest BUSCO (99.74%; 1515 out of 1519) and QV (57.73) scores among the gapless assemblies (Supplemental Table 3). Low-coverage regions (less than 5× coverage) 5 bp and longer were manually inspected and corrected by comparing read coverage across the corresponding region in other high-ranking assemblies, inspecting the raw reads for discrepancies in alignment to the assembly, and aligning available reads to each other to help determine the local consensus (Supplemental Table 4). Reads were then realigned, and the edited regions were evaluated to determine whether improvements had been made to the region. Many of the changes were obvious and were fixed by simple deletion or insertion of nucleotides based on the consensus of the raw reads. Alternate assemblies that had better read coverage across specific regions were used to replace sequence. We were unable to improve read coverage in four regions located on chromosomes 1, 2, 4, and 5, and these were unchanged but are noted in Supplemental Table 4. Small errors such as single base substitutions and local expansions or collapse errors were fixed by running Inspector (Chen et al., 2021b) (Supplemental Table 2), which detects sudden changes in read coverage to identify and correct small-scale errors. Previously mentioned metrics for completeness and accuracy were again checked before accepting any changes. The final telomere-to-telomere assembly size is 113 900 589 bp. Half of the whole assembly is contained in eight contigs that are each longer than 6 857 676 bp. BUSCO produced an initial score of 1515 out of 1519 using Augustus (Stanke and Morgenstern, 2005). Three of the missing BUSCOs were located by running BUSCO with MetaEuk (Levy Karin et al., 2020), and the last was recovered by comparing the expected amino acid sequence with aligned gene models. Merqury produced a QV score of 59.38 or a per-base error rate of 1.15 × 10^−6^. All bases in the assembly are supported by either HiFi or Nanopore reads, except for 4 bp at the left telomere of Chr10 and 1 bp at the right telomere of Chr12. At least 99.997% of the bases are covered by more than five reads from either HiFi or Nanopore (Supplemental Table 5).

### CC-5816 haplotypes, repeat content, cytosine methylation, and gene content

We characterized general genomic features of CC-5816. Using Illumina whole-genome sequencing paired-end reads from the CC-5816 parental strains CC-124 and CC-125, we created a haplotype map for each chromosome by comparing haplotype specific k-mers (hapmers) between the read sets and the genome assembly. We used the distribution of these hapmers to assign phase blocks and show that 74% of the haplotype derives from CC-124, whereas only 17% originates from CC-125 (Figure 2A). The nucleotide content of the assembly remains unchanged from previous estimates; it contains 64% G and C bases. The genome-wide repeat contents determined by RepeatMasker and Tandem Repeats Finder differed greatly, probably owing to their methods of detection (Supplemental Table 6). RepeatMasker uses an established database that includes annotated TEs and repeat sequences for comparison against the query sequence. It estimated a repeat content of 18.88%. Tandem Repeats Finder, which uses a non-biased approach to detect stretches of tandemly repeating sequence, estimated a repeat content of 7.72%. The union of these two programs together masked 22.37% of bases as repetitive sequence across the genome. Using the union of these results, we found that chromosomes 11 and 15 possessed strikingly high repeat contents: 34.34% and 55.71%, respectively. Chromosomes 4 (27.48%) and 5 (25.59%) have higher averages as well. Examining the spatial organization of the repetitive elements on chromosomes 11 and 15 revealed that they are not distributed equally across each chromosome. Instead, they fall within concentrated regions roughly 860 kb and 1.6 Mb in length, respectively, next to their respective centromeres (Figure 2B and 2C). 83.68% of the 1.6-Mb region on chromosome 15 was flagged as repetitive, and the 860-kb region on chromosome 11 contains 100% repetitive bases. It is likely to be the most repetitive region in the genome. Both regions are partially missing in alignments to both the CC-1690 and CC-503 v5 assemblies. Inspection of read coverage across these regions in CC-5816 did not reveal any evidence of mis-assemblies, expansions, or other errors; read coverage was consistent throughout the regions.

Using Nanopore reads, we assessed the frequency of CpG sites methylated to 5-methylcytosine (5mC) using Nanopolish, which performs well based on benchmarking studies (Simpson et al., 2017; Liu et al., 2021; Yuen et al., 2021). We found that average per-site 5mC frequencies for individual chromosomes range between 1.36% and 3.97%, with an average genome-wide frequency of 2.13%. This value falls between the two previous estimates of 0.75% (Lopez et al., 2015) and 5.38% (Feng et al., 2010), which were based on whole-genome bisulfate sequencing. Potential reasons for the range in genome-wide averages may include the life stage at which the cells were analyzed or the bioinformatic tools used to analyze the sequencing data (Tang et al., 2012; Simpson et al., 2017; Yuen et al., 2021). Despite the difference in average genome-wide 5mC frequency, locations of individual hypermethylated regions identified by Lopez et al. were validated by our Nanopore dataset. These hypermethylated sites predominantly overlap centromeres (Figure 2A) and other repetitive regions that are largely missing or incomplete in previous assemblies. These include the previously mentioned repetitive region on chromosome 11 (Figure 2A and 2B). Centromeres were identified on all 17 chromosomes by the presence of *ZeppL-1_cRei* LINE TE clusters (Lin et al., 2018; Craig et al., 2021) (Supplemental Table 7). Based on the length of *ZeppL-1_cRei* clusters, centromeres in CC-5816 range in length from approximately 252 to 480 kb. We saw a 15-fold enrichment in average per-site 5mC frequencies at CpGs across all centromeres (23.55%) compared with other nuclear CpGs (1.55%). For individual chromosomes, the average 5mC frequency across centromeres ranges between 4.26% and 34.53%. Regions annotated as repetitive sequence by RepeatMasker or Tandem Repeats Finder are also hypermethylated compared with non-repetitive regions (4.67% versus 1.42%). We mapped CC-503 gene models (Gallaher et al., 2021) to CC-5816 and observed that coding sequence (CDS) is hypomethylated relative to non-coding sequence (0.98% CDS; 3.11% non-coding). We then examined 5mC frequencies in the chloroplast and mitochondria. Previous studies have shown high methylation frequencies in chloroplast DNA derived from cells undergoing gametogenesis, especially in mating-type plus gametes (Feng and Chiang, 1984). We found that the frequency of methylation across the chloroplast derived from CC-5816 gametes is 24.52%, more than double the previous estimate made from mating-type plus gametes. The mitochondrial genome was methylated at a much lower frequency than the chloroplast in gametes (3.96%). Previous attempts to detect methylation from the mitochondrial genome of *Chlamydomonas* were unsuccessful (Beckers et al., 1991). To our knowledge, this is the first report of mitochondrial 5mC methylation in *Chlamydomonas*.

We used a combined dataset composed of Iso-Seq data derived from CC-124 mRNA and a publicly available multi-isolate Iso-Seq dataset (Gallaher et al., 2021) to identify potentially novel splicing events and gene loci. We compared gene models generated from Iso-Seq with v5.7 models (Supplemental Table 8). Six of the 19 548 v5.7 transcripts could not be mapped initially to CC-5816 and were checked for potential mis-assemblies surrounding adjacent genes. Cre12.g532400.t1.2 and Cre16.g650850.t1.2 are annotated as Tam3-transposase and are probably a strain-specific insertion. Cre12.g539700.t1.2 is redundant with two other genes that are located next to each other; they are Cre04.g229450 (e-175) and the combination of Cre04.g229398 and Cre04.g229422, which Iso-Seq reads suggest is one transcript. Cre11.g467557.t1.1 is found near a large gap in v5 and is split when aligned to CC-5816. Iso-Seq reads suggest that the two split parts of this model in CC-5816 are from the 5′ and 3′ ends of Cre11.g467551 and Cre11.g467552, respectively. Both models flank an assembly gap in v5. Cre17.g703300 is annotated as a ribonuclease III and has a high hmmsearch score to PlantFAM Chlorophyte protein family regulator of Ty1 transposition protein 103 (e = 2.8e−65) that includes the nearby gene Cre17.g703450 (e = 4e−27). Cre16.g655430 is an unannotated, green-algae-specific gene with no domain predictions. It is in a region on Chr17 that is highly variable between CC-125 and CC-124. This transcript is likely to be missing in CC-5816 owing to the difference in haplotype between the CC-5816 and CC-503 genomes. Read coverage from both HiFi and Nanopore was normal across each of these loci. We conclude that these six transcripts are strain specific, redundant, nonessential, or TEs and do not suggest mis-assemblies in CC-5816. A total of 78 827 Iso-Seq transcript models were generated, corresponding to 12 711 unique genes. SQANTI3 (Tardaguila et al., 2018) was used to compare Iso-Seq models with RNA sequencing (RNA-seq) data and v5.7 transcript models by comparing overlaps of internal splice sites, matching 10 935 v5.7 genes to 11 561 Iso-Seq genes. A total of 29 968 Iso-Seq transcripts precisely matched a v5.7 model, 16 827 Iso-Seq transcripts excluded exons and/or retained introns but used known splice sites, 27 344 contained novel splicing events, and 1150 Iso-Seq gene models were classified as novel. It is likely that some portion of these novel genes that overlap a v5.7 gene correspond to models that could not be matched by automated means; 110 of these novel genes contain transcripts that overlap a v5.7 model, each reciprocally covering at least 60% of the other’s length, and 174 show no overlap with a v5.7 model. Of the v5.7 genes, 6835 did not match an Iso-Seq model, and 5956 of these did not contain transcripts with overlap to the Iso-Seq datasets, indicating that detectable transcription is presumably not present for all genes in the combined Iso-Seq dataset. We examined transcripts that were present in the centromeric regions (Supplemental Table 9). Assuming an even distribution of genes with approximately one gene every 10 kb based on the number of Iso-Seq gene loci and genome size, 350 genes were expected. We found only 51 Iso-Seq gene models that mapped to a centromeric region, eight of which had only a very small overlap with the centromere boundaries (Supplemental Table 10). Chromosomes 6, 15, and 16 each contain six Iso-Seq gene models, 4 and 11 contain none, and the average number of genes is three. We performed a multiple sequence alignment of the 557 centromere transcript models, which revealed 80 separate clusters of transcripts with greater than 60% identity to each other (Supplemental Table 9). To determine the composition of each cluster, we took a random transcript from each cluster and performed a BLAST search, as well as examining RepeatMasker annotations within the gene models (Supplemental Table 10). Thirty-nine gene models were identified as likely TEs, the majority being Long inserted nuclear elements (LINEs). Four were unidentifiable, but each is similar to four unannotated genes (Cre02.g093300, Cre07.g333850, Cre09.g399812, and Cre14.g622550).

### Organellar sequences are present throughout the nuclear genome of CC-5816

Gene transfer between host and endosymbiont has played a large role in eukaryotic genome evolution (Martin and Herrmann, 1998). Organellar sequences continue to be identified in sequenced eukaryotic nuclear genomes, and there is growing evidence that mitochondrial and plastid DNA continues to insert into nuclear sequence and may play a role in human diseases, including cancer (Wei et al., 2022). Two independent groups observed *de novo* integrations of plastid DNA (NUPTs) in the tobacco nuclear genome (Huang et al., 2003; Stegemann et al., 2003). NUMTs have been observed and their frequency increased by mutations in *Saccharomyces cerevisiae* (Thorsness and Fox, 1990, 1993). Similar experiments to those performed in tobacco have been attempted in *C. reinhardtii* using a drug-resistant gene inserted into the chloroplast that confers resistance only if it is translocated to the nucleus (Lister et al., 2003). However, no evidence of active DNA transfer was detected, and no apparent plastid sequences were identified in the genome assembly. Several subsequent studies have found varying levels of organellar sequence spread throughout the genome, depending on the BLAST parameters used for the search and the version of the genome assembly available at the time (Richly and Leister, 2004; Smith et al., 2011). Using BLASTn with conservative parameters (see section “methods”), we found 33 NUPTs and 23 NUMTs ranging between 87.8% and 100% identity to the aligned organellar sequences (Figure 3A; Supplemental Table 11). All insertions are individually short in length, ranging from 28 to 384 bp. HiFi and ONT coverage across these elements and the surrounding context did not reveal any evidence of mis-assemblies (Figure 3B and 3C). To assess the likelihood of obtaining false positives, we used two independent methods. First, we randomized 17 sequences with 64% GC nucleotides of equivalent length to the *Chlamydomonas* chromosomes, each containing 64% GC nucleotides. BLASTn did not find any positive results from these random sequences. Second, each insertion was used as a query for BLASTn against the NCBI database (Supplemental Table 12). The most common result for each sequence was alignment to the respective *Chlamydomonas* organelle; there were a few hits to other organisms. These two tests provide strong evidence for the presence of organellar DNA in the nuclear genome.

We characterized the NUPTs and NUMTs. Each chromosome has at least one NUPT or NUMT (Figure 4A). To determine whether the locations of NUPTs or NUMTs have any correlation with other features in the nuclear genome, we looked at the distribution of these elements with respect to annotated repetitive elements, genes, nucleotide content, and methylation frequency (Figure 4B). We generated 100 sets of shuffled NUPTs and NUMTs of similar size for each analysis to compare against a random distribution. We considered all annotated features within 50 bp of NUPTs and NUMTs in our analyses. We saw differences in the frequency of various genomic elements appearing in proximity to tested sites depending on whether the tested site was an NUPT, NUMT, or random site. Repetitive elements in general were underrepresented adjacent to NUPTs (RepeatMasker, *p* = 0.01; Tandem Repeats Finder, *p* = 0.03). Specifically, we found simple repeats to be statistically underrepresented adjacent to NUPTs (*p* = 0.04). Despite the underrepresentation of repetitive elements, helitron transposons were near NUPTs more frequently than expected by chance (*p* = 0.03). NUMTs, on the other hand, did not appear to have any significant correlation with any repetitive elements, aside from possibly low-complexity regions, the element nearest to our *p-value* threshold of 0.05 (*p* = 0.1). We then looked at the proximity of NUPTs and NUMTs with respect to v5.7 genes and saw that NUMTs were preferentially located near intronic regions and UTRs (*p* = 0.02), whereas NUPTs did not appear to have any correlation with genes. The average GC contents of chloroplast and mitochondrial sequences are very different from that of the *Chlamydomonas* nuclear genome (34.57% for chloroplasts and 45.24% for mitochondria). Therefore, it is not surprising to find that sequences putatively originating from these organelles are, on average, lower in GC content than the nuclear genomic average of 64% (NUPTs, 43.8% GC, *p* = 6.64 × 10^−18^; NUMTs, 49.1% GC, *p* = 7.76 × 10^−9^). We looked at the 50 bp flanking each insertion and found that, to a lesser degree, GC content was lower than expected (NUPTs, 59.2% GC, *p* = 6.64 × 10^−18^; NUMTs, 58.8% GC, *p* = 7.76 × 10^−9^). Several studies have shown that organellar insertions in nuclear DNA are often hypermethylated, especially before accumulating mutations (Yoshida et al., 2019; Fields et al., 2022). We observed a small, but not statistically significant (*p* = 0.28), enrichment of 5mC frequencies at NUPT CpGs (4%) compared with non-NUPT CpGs (2.1%). We next asked whether NUPTs or NUMTs derive from specific regions within the chloroplast or mitochondrion (Figure 4C). For each NUPT and NUMT, we used the BLAST alignments with the highest identity to determine the likely origin of the donor. Insertions with more than one equally likely donor were included in the analyses. We again randomly sampled 100 sets of coordinates of equal size to the actual donor sequences from the chloroplast and mitochondrion for comparison. For NUPT donors within the chloroplast, we saw an underrepresentation of annotated genes within 50 bp of donor sequences (*p* = 1.2 × 10^−3^) and even more specifically with coding sequence (*p* = 4.1 × 10^−9^). We found NUPT donor regions enriched for 3′ UTRs (*p* = 8.9 × 10^−4^), rRNA sequence (*p* = 9.9 × 10^−6^), and GC nucleotides (donor, *p* = 9.9 × 10^−6^; 50 bp flanking, *p* = 9.9 × 10^−6^). We then calculated the distance of each donor to its nearest neighboring donor to determine whether there was any clustering. The mean distance between each donor and the nearest neighboring donor is shorter than in any of our random samples by an average of ∼317 bp (*p* = 3.9 × 10^−3^). We identified clustering of NUPT donors within the two inverted repeat regions (*p* = 1.6 × 10^−5^) and observed a weak signal for NUPTs clustered near elements associated with mobile DNA, namely group I and group II introns (Novikova and Belfort, 2017), which were closer to NUPTs on average, although this association was not statistically significant in our tests (group I introns, *p* = 0.09; group II introns, *p* = 0.11).

### Ribosomal DNA arrays

**Figure 5:**
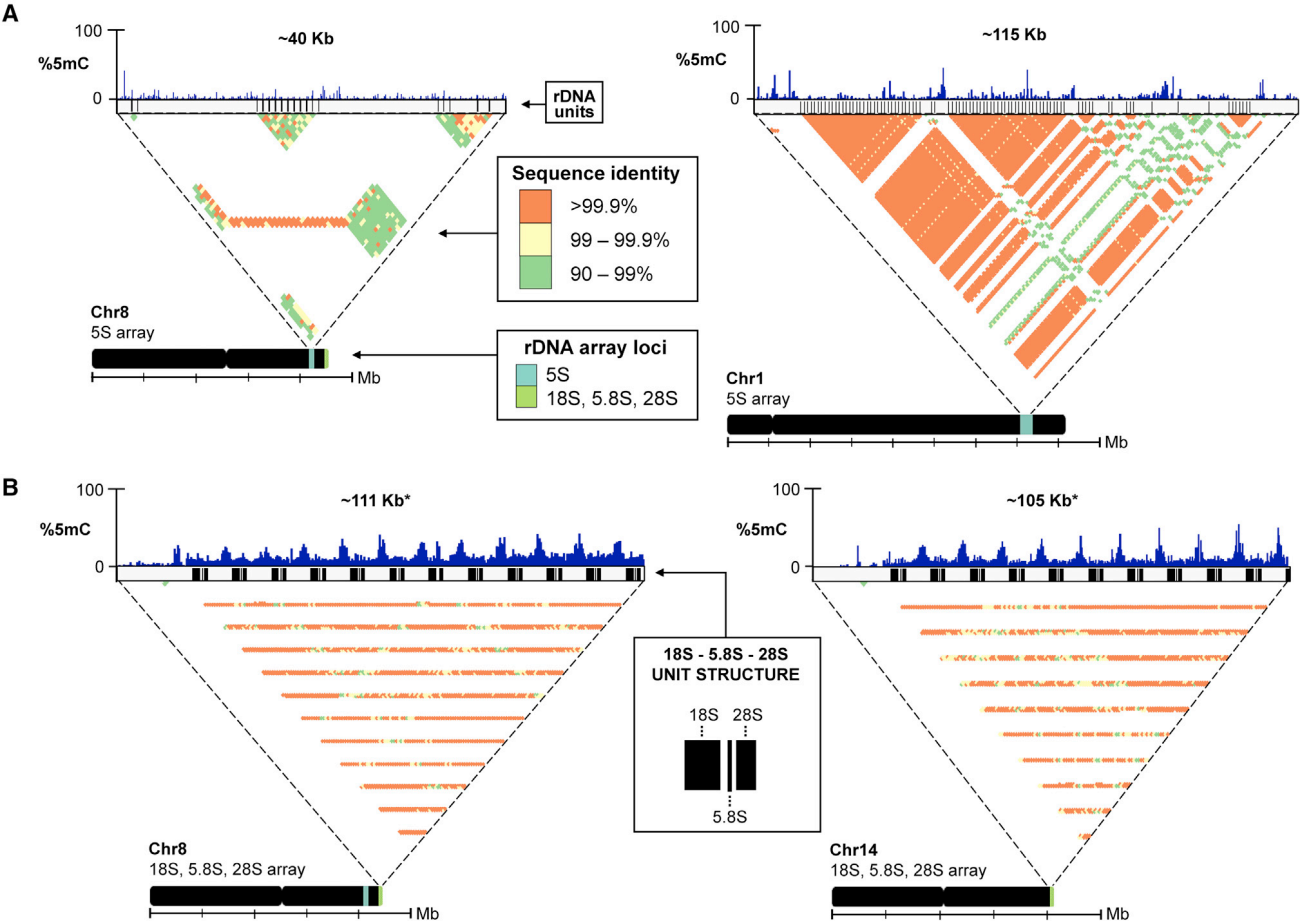
rDNA arrays. **(A)** Characterization of 5S arrays on chromosomes 8 (left) and 1 (right) with approximate length of the region in the assembly, 5mC frequency, unit structure, percentage identity heat map, and ideogram illustrating location of arrays (5S, light blue; 18S, 5.8S, and 28S, lime green). **(B)** Characterization of 18S, 5.8S, and 28S arrays on the ends of chromosomes 8 (left) and 14 (right). The sequence identity and array loci legends in **(A)** apply to **(B)** as well. Asterisks (∗) indicate length in the final genome assembly.

We next looked at the location of ribosomal DNA (rDNA) in CC-5816. There are four main arrays in CC-5816, two 5S arrays on chromosomes 1 and 8 and two 18S, 5.8S, and 28S arrays on chromosomes 8 and 14 (Figure 5A and 5B), as well as ∼12 singular or partial rDNA units scattered throughout the rest of the genome. The 5S array on chromosome 8 was completely assembled using only HiFi reads (Figure 5A). The 5S array on chromosome 1 is ∼3× longer with highly identical rDNA units (Figure 5A). This array was only correctly assembled after incorporation of Nanopore reads. Consistent with observations from strain CC-1690 (Chaux-Jukic et al., 2021), 18S, 5.8S, and 28S rDNA are located on the right arms of chromosomes 8 and 14 and are organized tandemly in an array (Figure 5B). Based on alignment of PacBio Iso-Seq data, one 18S, 5.8S, and 28S unit is ∼6.46 kb in length and followed by a ∼1.98-kb spacer sequence to create a periodicity of ∼8.42 kb. The 18S, 5.8S, and 28S arrays are likely incomplete in length based on higher coverage than the genomic average and the presence of 85 additional contigs containing only these arrays.

### Gene editing of the *PF23* gene by CRISPR-Cas9

**Figure 6:**
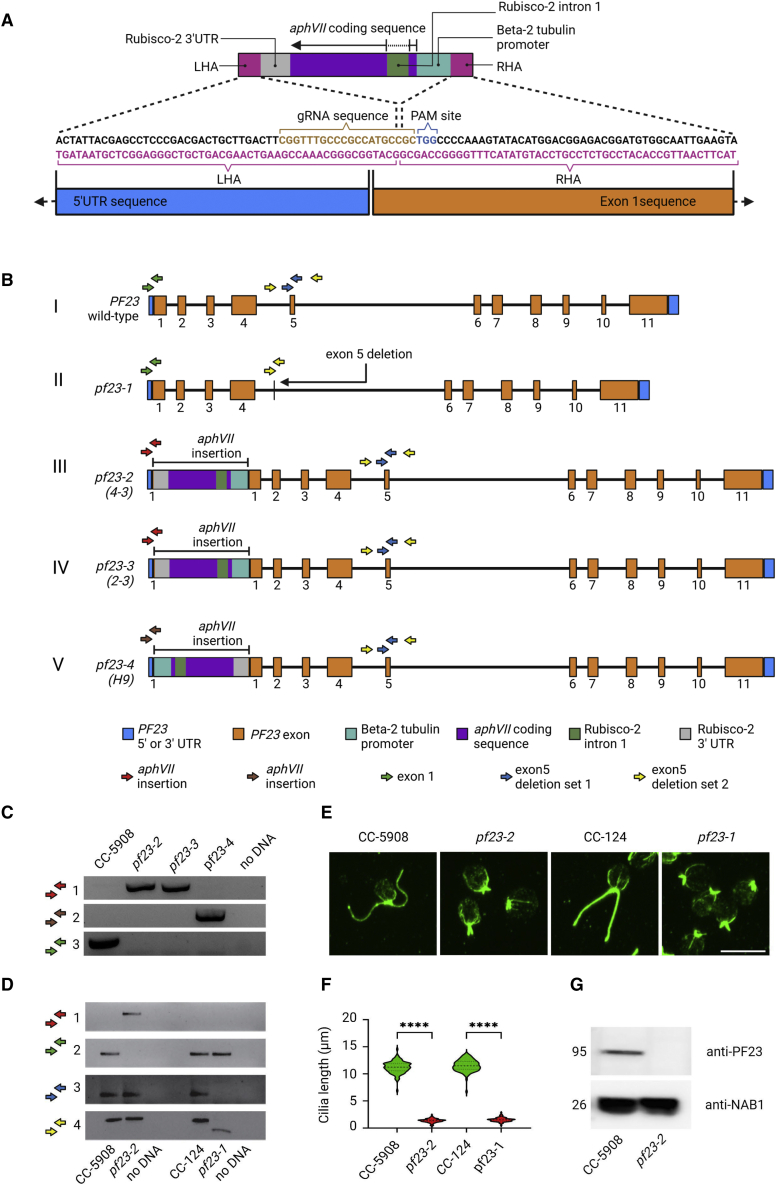
Characterization of a null *pf23* CRISPR-generated allele. **(A)** The *aphVII* construct includes the Beta-2 tubulin promoter, Rubisco-2 3′ UTR, and *aphVII* coding sequence with Rubisco-2 intron 1 sequence. The construct is flanked at the left and right ends with a left homology arm (LHA) and right homology arm (RHA) that correspond to regions in the 5′ UTR and exon 1 segments of the *PF23* gene. The dashed lines indicate that the homology arms in the *aphVII* construct recombine with the homologous sequences in the 5′ UTR and beginning exon 1 sequences of the *PF23* gene. Only a portion of the gene is shown for reference. The 5′ UTR and exon 1 sequences extend to the left and right, respectively, indicated by black arrows. The LHA and RHA sequences are in pink, the guide RNA (gRNA) in tan, and the PAM site in blue. Cas9 cleavage at the PAM site allows a break in the *PF23* gene followed by recombination of the RHA and LHA to allow insertion of the *aphVII* construct into exon 1 of *PF23*. Maps generated to scale using BioRender. **(B)** Colored arrows indicate regions of PCR amplification by primers listed in Supplemental Table 13. (I) The wild-type *PF23* gene. (II) The *pf23-1* allele lacks exon 5 and portions of the flanking introns, as indicated by the black arrow. (III) The *pf23-2* strain is expected to contain an insertion of the *aphVII* construct in reverse orientation within exon 1 (bars indicate region of insert ). GreenGenie2 was used to annotate the correct *PF23* gene using Nanopore sequencing assembly (this study) and a cDNA sequence of *PF23* (Yamamoto et al., 2017). (IV) The *pf23-3* strain also contains an insertion of the *aphVII* construct in reverse orientation. (V) The *aphVII* insertion in the *pf23-4* strain is in the forward orientation. Maps generated to scale using BioRender. **(C)** PCR to detect *aphVII* hygromycin insertion in *pf23* CRISPR-generated strains. Primers in exon 1 and *aphVII* (*PF23-aphVII* junction) show that *aphVII* has inserted in the reverse orientation in *pf23-2* and *pf23-3* (row 1, red arrows), whereas *aphVII* is present in the forward orientation in *pf23-4* (row 2, brown arrows). Primers spanning the exon 1 CRISPR insertion site only amplify in wild-type strain CC-5908 that lacks the insertion (row 3, green arrows). **(D)** Comparison of the mutations present in *pf23-2* and *pf23-1* strains. Primers spanning the *PF23*-*aphVII* junction amplify only in the *pf23-2* strain containing the insertional construct (row 1, red arrows). Primers flanking the CRISPR site in exon 1 fail to amplify in *pf23-2* owing to the large insertion and use of a short extension time during PCR (row 2, green arrows). Primers within exon 5 fail to amplify in *pf23-1* that contains a deletion of exon 5 and parts of the surrounding introns (row 3, blue arrows). This deletion is absent in the *pf23-2* CRISPR-generated strain and wild type; those strains both amplify PCR fragments that are 955 bp long (row 4, yellow arrows), whereas *pf23-1* generates a 494-bp fragment. Colored arrows indicate primer binding sites in **(A)**. **(E)** Immunofluorescence of strains with wild-type length cilia (CC-5908) and CC-124 compared with CRISPR-generated strain *pf23-2* and a strain containing the *pf23-1* allele that have short cilia. The scale bar corresponds to 10 μm. **(F)** Quantification of cilia length of strains. One hundred individual cilia (one per cell) were measured for each strain. Asterisks indicate *p* <0.0001 using an independent two-sample two-tailed *t*-test. **(G)** Immunoblot of the *pf23-2* strain shows that *PF23* protein is completely absent from this strain. NAB (Nucleic acid binding) protein 1 is used as a loading control.

The *PF23* gene contains 11 exons (Figure 6B, I) that encode a dynein assembly factor (DNAAF) protein, which is needed for assembly of axonemal dynein arms in the cilia. It is known as DXY1C1/DNAAF4 in mammals (Tarkar et al., 2013). The only allele of the *PF23* gene in *Chlamydomonas* (*pf23-1*) contains an in-frame 494-bp deletion that removes all of exon 5 and parts of the two flanking introns (Figure 6B, II). This generates a protein lacking 27 amino acids (Yamamoto et al., 2017). Although this allele has been extensively characterized, the study of null alleles often provides further insights into function and could reconcile differences in phenotypes between *Chlamydomonas* and human variants in *DYX1C1*. We utilized a site-directed insertional mutagenesis approach using CRISPR-Cas9 to generate null alleles of *PF23*. A Cas9 gRNA protospacer adjacent motif (PAM) site at the beginning of exon 1 of the *PF23* gene was chosen, as mutations at this site would likely result in disruption of the gene. Cas9 protein, the gRNA construct, and an *aphVII* gene that confers resistance to hygromycin with reverse-oriented homology arms (Picariello et al., 2020) were transformed into either the CC-5908 or CC-5909 strain (Supplemental Table 16) to insert the *aphVII* gene into exon 1 (Figure 6A). Following transformation and selection on hygromycin medium, we isolated three independent strains (4-3, 2-3, and H9) that failed to swim in liquid cultures, which is the phenotype of the *pf23-1* allele. PCR analysis using primer pairs upstream of exon 1 and within the *aphVII* construct (*PF23*-*aphVII* junction) showed that the *aphVII* construct was inserted into exon 1 of *PF23* in all three strains (Figure 6C; Supplemental Tables 13 and 14). The *aphVII* construct was inserted in the reverse orientation in strains 4-3 and 2-3, as expected (Figure 6B, III and IV and 6C, rows 1 and 2). However, in strain H9, *aphVII* was integrated in the forward orientation (Figure 6B, V and 6C, row 3). Figure 6D (row 1) shows that the *aphVII* insertion is present in *pf23-2* but not wild-type strains CC-5908 and CC-124 (see also Figure 6B, III). Primers flanking the exon 1 gRNA site failed to amplify in *pf23-2*, suggesting the insertion of a large fragment of DNA (Figure 6D, row 2). Our analysis also showed that the mutation in *pf23-2* was distinct from the original *pf23-1* allele that contains an exon 5 deletion (Figure 6D, rows 3 and 4; Supplemental Tables 13 and 14). To confirm that the short-cilia phenotype was linked to *aphVII* insertion into the *PF23* gene, we performed two backcrosses on each strain, followed by meiotic analysis. Analysis of seven tetrads from 4-3, 12 tetrads from 2-3, and 12 tetrads from H9 showed complete segregation of the short-cilia phenotype with hygromycin resistance and PCR amplification of the *PF23*-*aphVII* junction. These results confirm that the short-cilia phenotype resulted from disruption of the *PF23* gene. Consequently, strains 4-3, 2-3, and H9 were renamed *pf23-2*, *pf23-3*, and *pf23-4,* respectively. The *pf23-4* strain showed wild-type levels of viability in tetrads (*n* = 30). However, the seven tetrads from strain *pf23-2* and the 12 tetrads from strain *pf23-3* produced ∼50% inviable meiotic progeny. This suggests the presence of large chromosomal aberrations that cause aneuploidy and death following meiosis. This observation is investigated further in the section below on meiotic analysis. Immunofluorescence analysis showed that strain *pf23-2* was unable to grow full-length cilia. The cilia were short and stubby, resembling those of the original *pf23-1* strain (Figure 6E and 6F). To confirm that the *pf23-2* strain was a protein null, we performed immunoblot analysis with a polyclonal antibody against PF23 protein (Yamamoto et al., 2017). Figure 6G shows that the *pf23-2* strain completely lacks the PF23 protein.

### Meiotic analysis of *pf23* null CRISPR-generated strains

Tetrad analysis has been used extensively to analyze the meiotic outcomes in *Neurospora* and *Saccharomyces* strains that are heterozygous for a translocation, which reduces the viability of meiotic progeny. Examination of translocations in *S. cerevisiae* and *Neurospora crassa* by tetrad analysis shows that tetrads with four viable progeny, two viable progeny, and no viable progeny are the most common classes (Perkins, 1974; Loidl et al., 1998). Tetrads with four viable progeny are ascribed to alternate segregation that generates progeny with two wild-type chromosomes and two balanced translocation chromosomes. Tetrads with only two viable progeny are ascribed to alternate segregation with recombination between the breakpoint and the centromere, or nondisjunction. Tetrads with no viable progeny are ascribed to adjacent segregation with all progeny having unbalanced chromosomal complements.

**Figure 7:**
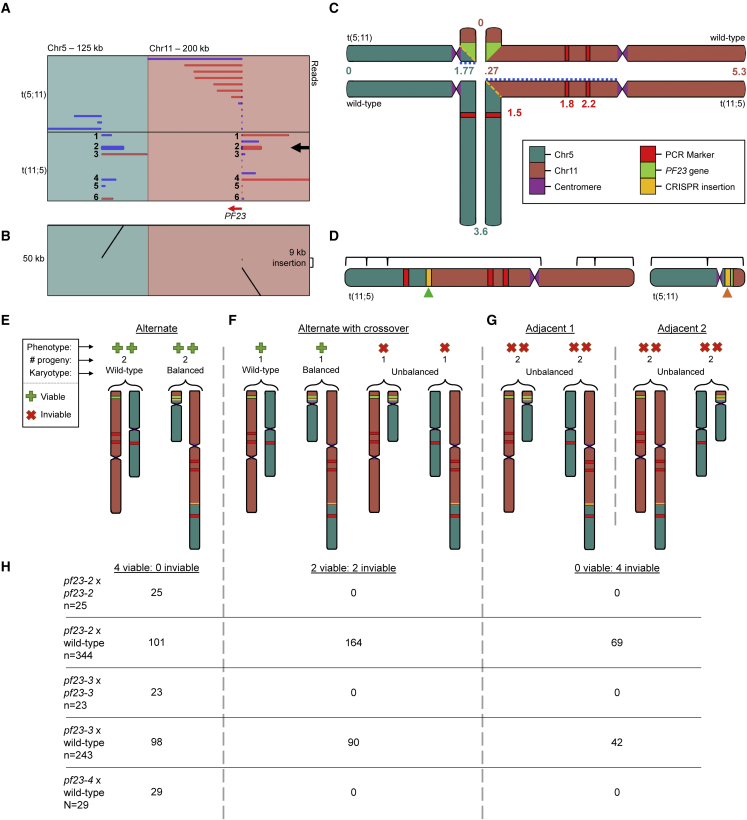
Translocations in *pf23-2* and *pf23-3* were induced by site-directed CRISPR-Cas9 mutagenesis. **(A)** Alignments to CC-5816 from *pf23-2* Nanopore reads selected within the *PF23* gene locus. Each row corresponds to a single aligned *pf23-2* Nanopore read. Blue and red lines along the x axis indicate forward and reverse alignments, respectively. The read in bold indicated by the black arrow highlights one example of a chimeric read mapped with respect to CC-5816 that overlaps the translocation break point in *pf23-2*. Alignments to chromosomes 2 and 12 are due to the RBCS2 intron and Beta2-tubulin promoter, respectively, which are used in the insertional cassette. **(B)** Dot plot of the bolded read in **(A)**. **(C)** Quadrivalent structure of Chr5, Chr11, t(5;11), and t(11;5) predicted to form during meiosis I in a *pf23-2* × wild-type cross. Similar information for *pf23-3* can be found in Supplemental Figure 6. Coordinates are in megabases and based on CC-5816 to illustrate approximate location of breakpoints in translocation. Alternate segregation of chromosomes containing a single crossover between the breakpoints and centromeres results in one-half of progeny being inviable owing to deletions in either chromosome. **(D)***pf23-2* Nanopore reads were assembled *de novo* using Flye as validation to determine k-mer overlapping of putative translocation junctions. Brackets indicate regions supported by an assembled contig. A contig spanning the entirety of a highly repetitive region downstream of the Chr11 centromere could not be found. The region denoted by the green arrow is supported by linkage of PCR-based markers shown in red, *de novo* genome assembly with Flye, and six individual reads spanning the entire breakpoint, including Chr5, the connecting *aphVII* insertion cassette, and Chr11. The breakpoint denoted by the orange arrow has reads from either Chr5 or Chr11 connected to an *aphVII* insertion cassette. *De novo* genome assembly with Flye was used to determine whether the breakpoint could be assembled *de novo* into a contig, indicating the presence of unique overlapping k-mers from these reads. **(E–G)** Diagram depicting possible meiotic products from meiosis **(C)** for **(E)** alternate segregation without a crossover (four viable progeny), **(F)** alternate segregation with a single crossover event between a break point and centromere (2 viable and 2 inviable progeny), and **(G)** adjacent-1 and adjacent-2 segregation (all inviable). **(H)** Table of the number of tetrads containing either 4 viable, 2 viable, or 0 viable progeny from the meiotic crosses in this study. The parent strains in each cross are shown in the leftmost column with the number of tetrads scored (*n*). Note that **(C)** and **(E–G)** contain pictures corresponding to chromosomes from *pf23-2* × wild-type crosses.

Meiotic inviability in strains *pf23-2* and *pf23-3* after mating to a wild-type strain suggests the possibility of large chromosomal aberrations/rearrangements in the genomes of these CRISPR strains. Meiotic crosses of *pf23-2* by *pf23-2* produced fully viable tetrads (*N* = 25), as did meiotic crosses of *pf23-3* × *pf23-3* (*N* = 23) (Supplemental Table 15). Moreover, meiotic crosses of *pf23-2* × *pf23-3* suggest that the rearrangements in these two strains are not identical, as the viability of the meiotic progeny from this cross was lower than that from either single strain; only four tetrads produced four viable progeny, and 12 tetrads produced no viable progeny (*N* = 20). Using our optimized HMW DNA extraction protocol (see section “methods”; Supplemental Figure 2), we performed whole-genome Nanopore sequencing of *pf23-2* and *pf23-3*. We obtained 1.24 Gb of sequence or 10× coverage for *pf23-2* and 0.69 Gb of sequence or 6× coverage for *pf23-3*. We used a combination of *de novo* genome assembly and mapping of reads from *pf23-2* and *pf23-3* to the CC-503 v5 and CC-5816 genome assemblies to identify structural variations between the two CRISPR-generated strains (Supplemental Figure 5). We determined that the *pf23-2* strain carries a translocation between chromosomes 5 and 11, referred to as t(5; 11) and t(11; 5) (Figure 7A–7C). Breakpoints at exon 1 of *pf23* on Chr11 were identified using both CC-5816 and CC-503 v5. However, we were only able to identify the precise location of the breakpoint on Chr5 using CC-5816, as CC-503 lacks 14 kb of sequence in this region (Supplemental Figure 5). The breakpoint on translocation partner t(5; 11) from strain *pf23-2* was not covered entirely by a single read, which indicates a large insertion. To resolve the length of the insertion, we assembled *pf23-2* Nanopore reads *de novo* using Flye (Figure 7D). The resulting assembly revealed the insertion size to be ∼22 kb (Supplemental Figure 5). We then determined that *pf23-3* carries a translocation between chromosomes 3 and 11, referred to as t(3; 11) and t(11; 3) (Supplemental Figure 6). Breakpoints in *pf23-3* were identified using both CC-5816 and CC-503.

Because the number of reads across the translocation breakpoints was not large, we verified the translocations with meiotic crosses by determining whether linkage was observed between the translocation chromosomes predicted from the Nanopore sequencing. We examined meiotic progeny from 344 tetrads from crosses of *pf23-2* by two different CC-125 × CC-124-derived meiotic strains named 1-1 and 3-3. We examined 243 tetrads from crosses of *pf23-3* by 1-1 or CC-124 (see section “methods”). As observed in fungal tetrads heterozygous for a translocation, we found tetrads with four, two, and zero progeny (Perkins, 1974; Loidl et al., 1998). From *pf23-2* crosses, we retrieved 101 tetrads with four viable progeny (Figure 7E), 164 tetrads with two viable progeny (Figure 7F), and 69 tetrads with no viable progeny (Figure 7G). Tetrads with four viable progeny should show pseudolinkage of genes on the two translocated chromosomes. This means that SNVs on chromosomes 5 and 11 will be linked to each other and will not show independent assortment. We monitored PCR-based markers (Figure 7H; Supplemental Table 15) on chromosomes 5 and 11 in control crosses of CC-125 × CC-124. The genes on chromosomes 5 and 11 show independent assortment and therefore are unlinked in 28 tetrads (Figure 7H; Supplemental Table 15). In *pf23-2* × 1-1 (or 3-3), we found no recombination in 90 tetrads that showed 2:2 segregation of hygromycin resistance, and three tetrads with recombination. Nine tetrads that did not show 2:2 segregation of the *pf23* phenotype were found and may have arisen by nondisjunction. Among the tetrads that produced two viable and two inviable progeny, 89% had one product with the *pf23-2* allele and one with the wild-type allele. We monitored recombination between the breakpoint and the centromere on chromosome 5 but were unable to find markers between the centromere and the breakpoint on chromosome 11 (Supplemental Table 15). We observed that 21% of the tetrads showed recombination and 11% showed nondisjunction based on the lack of 1:1 segregation.

The *pf23-3* mutant is associated with a translocation between chromosome 3 and chromosome 11. Crossing either *pf23-4* or *pf23-1* by *ac17* showed that the two chromosomes assort independently (14:15:0 and 13:17:0, respectively) and that both genes are linked to their respective centromeres, given the lack of tetratype tetrads. From *pf23-3* crosses by the wild type, we retrieved 98 tetrads with four viable progeny, 90 tetrads with two viable progeny, 18 tetrads with only one viable progeny, and 42 tetrads with no viable progeny. The class with only one viable progeny was more frequent in the *pf23-2* crosses. In tetrads with four viable progeny from the *pf23-3* × 1-1 or CC-124 crosses, we found no recombination in 98 tetrads with *ac17,* which was present in the parental strain (CC-5908). *AC17* encodes the pyruvate dehydrogenase E1 beta subunit and maps at 6.45 Mb on chromosome 3 (Dent et al., 2015). In the 32 tetrads that were tested and were heterozygous for a PCR marker at 7.42 Mb, we observed no recombinants. For the tetrads that produced two viable and two inviable progeny, 95.6% had one product with the *pf23-3* allele and one with the wild-type allele. The other four had one with four non-swimmers, one with four swimmers, one with one non-swimmer and four swimmers, and one with three non-swimmers and one swimmer. In the 32 tetrads that were tested and heterozygous for the PCR marker at 7.42 Mb on chromosome 3, we observed three recombinants. Of the 18 tetrads that had only one viable progeny, most were swimmers (*N* = 14), and four of these showed resistance to hygromycin, which suggests that they are aneuploid and carry the translocation with chromosome 11 where the cluster of *aphVII* genes are located near the centromere of chromosome 11 (Supplemental Figure 6).

### Behavior of other CRISPR-generated strains in meiosis

Because we found that two of the three CRIPSR/Cas9 mutants in the *PF23* gene had translocations, we tested CRISPR-generated mutants in three other genes. We obtained a CRISPR-Cas9-generated strain with mutant alleles in *CHR1* and *CHR2* that encode channelrhodopsins (Greiner et al., 2017), as well as four mutant alleles in *FAP70* whose encoded protein is found in the central apparatus of cilia (Picariello et al., 2020). Each was crossed by a wild-type strain, and viability patterns were assayed. The generation of two viable and two inviable progeny, as well as no viable progeny, was considered indicative of a chromosomal aberration. The *chr1; chr2* strain produced 94% viable progeny (*N* = 18) and was judged to be structurally intact. The four *fap70* strains showed reduced viability that ranged from 30% to 50% (*N* = 82 tetrads). In a second backcross, the viability remained lower than that observed in the *pf23-2* and *pf23-3* crosses, which had 67% and 59% viability, respectively. However, three of the four *fap70* strains showed patterns of meiotic inviability suggestive of a chromosomal aberration (Supplemental Table 15). In the small subset of mutants tested, insertions in *fap70* and *pf23* but not in *chr1* and *chr2* showed signs of a chromosomal aberration. Thus, five of nine CRISPR-generated insertions were associated with a translocation or a suspected translocation.

## Discussion

A growing number of genetic and genomic tools are accessible to researchers; these include the telomere-to-telomere version of the human genome (T2T-CHM13) (Nurk et al., 2022) as well as CRISPR-Cas9-based methods for gene editing (Cong et al., 2013; Jinek et al., 2013). The community studying the widely used model organism *C. reinhardtii* has quickly adapted and produced similar tools tailored to this model organism. These include RNA-seq experiments (Albee et al., 2013; Zones et al., 2015; Joo et al., 2017; Gallaher et al., 2021), epitope tagging of proteins (Emrich-Mills et al., 2021), an insertional mutant library (Li et al., 2019), and CRISPR-Cas9 site-directed mutagenesis techniques (Shin et al., 2016; Picariello et al., 2020; Akella et al., 2021). These tools require a better reference genome assembly than the one assembled using short reads (Merchant et al., 2007; Blaby et al., 2014). Our telomere-to-telomere assembly of the 17 chromosomes of CC-5816 provides a complete reference for non-coding sequences, repetitive elements, and TEs, as well as DNA methylation. Another recent genome assembly (Craig et al., 2022) has been generated, but it has several gaps. Multiple genome sequences will be important for further genetic studies.

We evaluated various bioinformatic tools through stepwise assessment of multiple assemblies to determine which tools are most effective at generating high-quality genome assemblies from high-coverage HiFi and Nanopore sequencing data. Using ∼150× HiFi data, hifiasm generated the most contiguous and accurate nuclear assembly, whereas Canu generated mis-assemblies in multiple regions. TGS-GapCloser and QuickMerge were tested, and both were successful in producing high-quality assemblies. The order of assemblies used for gap filling with QuickMerge greatly affected the quality of the output, and careful evaluation should therefore be performed. We found the use of RaCon was detrimental to the quality of the assembly when used for polishing the entire assembly. However, using RaCon to correct short regions with low coverage improved the base quality score. For ∼50× Nanopore data, we evaluated three different assemblers (Canu, Flye, and NECAT) and found all three programs to be useful for comparing strategies for filling gaps and correcting regions with low coverage, as each varied in quality within different regions based on read coverage. Notably, Flye was the only assembler to generate a contiguous assembly (135 contigs and an N50 of ∼2.2 Mb) from low-coverage Nanopore data (<10× coverage).

### Regions resolved by long reads

Cumulatively, only 2916 bp of our assembly has less than 5× coverage from both HiFi and Nanopore reads. Some DNA segments in CC-5816 are unaligned to CC-503 v5.6 or CC-1690. Considering only one-to-one alignments, there are 8 164 010 bp and 4 488 839 bp in CC-5816 with no alignments to CC-503 v5.6 or CC-1690, respectively. Most of this sequence is flagged as repetitive and resides on chromosomes 11 and 15. The alignment of Iso-Seq reads suggests that ∼300–500 kb of this repetitive sequence may be transcriptionally active. Excluding repetitive DNA, there is a total of 1 999 696 bp and 932 474 bp of unaligned sequence compared with CC-503 v5.6 and CC-1690, respectively. It will be interesting to see the extent of genomic variation among *Chlamydomonas* strains upon release of more high-quality assemblies.

The identification of a ciliary central apparatus-associated protein from single particle cryo-electron microscopy (cryo-EM) of *Chlamydomonas* axonemes was made possible with the new assembly (Gui et al., 2022). In the v5.7 assembly, *FAP221* (Cre11.g476376) and *FAP360* (Cre49.g761347) are currently annotated as two different gene models. Nucleotide and amino acid sequence alignments to CC-5816 as well as Iso-Seq models suggest that *FAP221* and *FAP360* are not paralogs, but are actually one gene (Supplemental Figure 7). Based on Iso-Seq transcript models, 2435 transcripts match splice junctions from more than one v5.7 gene model, indicating that there may be many gene models in need of correction. This will be a key area for future work.

Based on previous studies, there are roughly 350 copies or 3 Mb of rDNA genes in the genome (Howell, 1972; Marco and Rochaix, 1980; Chaux-Jukic et al., 2021). However, our assembly contains only 12 full rDNA copies on chromosome 8, and 10 full and one partial copy on chromosome 14. Assembling repetitive arrays of this size remains challenging even with long reads. We have turned to assessing rDNA number using HiFi and Nanopore coverage across rDNA arrays compared with genome-wide coverage. We estimated that there are 2 758 766 to 2 809 246 bp of total rDNA sequence, which corresponds to 328–334 units. Using read coverage for each chromosome separately, we found that the chromosome 8 array has 1 393 494 bp of sequence, or 129 copies, based on HiFi and 658 050 bp, or 78 copies, based on Nanopore. The chromosome 14 array has 1 667 328 bp of sequence, or 198 copies, based on HiFi and 2 140 753 bp, or 245 copies, based on Nanopore. Thus, our coverage data support the previous studies.

Coverage from our long-read datasets provides evidence for 56 sites of organellar DNA insertions into the nucleus (Supplemental Table 11). Previous work using short reads observed AT-rich sequence thought to be plastid in origin linked to telomere sequences (Flynn et al., 2018). We observed plastid sequences linked to telomere sequence on chromosomes 2, 3, 5, 9, and 17, and mitochondrial sequence on the chromosome 14 telomere supported by <5 reads. A contig with mostly plastid sequence containing ∼2 kb of nuclear sequence was supported by one read. BLAST aligned the nuclear portion of this contig to 12 genes (Supplemental Table 17). Thus, the evidence for telomere-linked plastid sequence is marginal.

Many eukaryotes modify cytosines in CpG dinucleotides by the addition of a methyl group at the C-5 position. This modification has a multitude of functions that include transcriptional repression or maintenance of genomic stability (Baubec and Schübeler, 2014; Nasrullah et al., 2022). Methylation is not utilized to the same degree in all eukaryotes. For example, yeasts display very low levels of 5mC (<0.4%), and this varies depending on the life stage (Tang et al., 2012). Using detection of base modifications in Nanopore reads, we found that the average genome-wide 5mC frequency in *C. reinhardtii* was 2.13% in gametic CC-5816 cells. This average falls between two previous estimates (0.75% and 5.38%) based on whole-genome bisulfite sequencing (Feng et al., 2010; Lopez et al., 2015). Technical explanations for this variation include differences in sequencing technologies, bioinformatics pipelines, or the genome assembly used for read alignment. We chose to use Nanopolish, which uses a hidden Markov model to detect base modifications from Nanopore reads. Benchmarking studies have demonstrated the accuracy of Nanopolish in detecting modifications across a broad range of methylation frequency contexts, although one caveat to consider is that it has been shown to sometimes overestimate methylation in low-5mC-frequency regions (Liu et al., 2021; Yuen et al., 2021). Biological explanations include differences in strains or life stage. Feng et al. (2010) reported a genome-wide methylation frequency of 5.38% in vegetative cells from the *MT+* strain CC-503, using the CC-503 v3 assembly for read alignment. Lopez et al. (2015) used a different strain and the CC-503 v5 assembly. They detected 5mC frequencies <0.75% using both plus and minus mating-type strains and saw little difference between zygotic, gametic, and vegetative cells.

Centromeres in many organisms are hypermethylated (Fachinetti et al., 2015; Naish et al., 2021). DNA methylation appears to be important for binding of the centromere-specific histone H3 (CenP/CEH3). Our data show that the *Chlamydomonas* centromeres are highly methylated compared with the rest of the chromosomal arms. Centromeres are required for chromatid cohesion and segregation during meiotic and mitotic cell divisions. It will be interesting for future studies to ask about the rates of chromosome loss and meiotic recombination on different chromosomes with varying amounts of DNA methylation (Miller et al., 2005).

### Translocations induced by CRISPR-mediated mutagenesis

Both insertional and targeted base-pair alterations have been attempted using a variety of techniques in conjunction with CRISPR-Cas9. Gene editing is associated with the generation of tandem arrays. The *aphVII* gene was inserted into *pf23-2* and *pf23-3* in tandem arrays that contained both complete and truncated copies. We found 14 and nine full-length copies of *aphVII* in *pf23-2* and *pf23-3*, respectively. This phenomenon has been reported in insertional mutagenesis of *Schizosaccharomyces pombe,* in which tandem repeats of *ura4*^*+*^ transgenic DNA were inserted at a single site by non-homologous end-joining (Davidson et al., 2004). Another group reported similar results after transformation of *Magnaporthe oryzae* using cassettes that contained the hygromycin gene (*aphVII*) (Betts et al., 2007). In *C. reinhardtii,* this occurrence has also been observed following insertional mutagenesis of the nuclear genome (Nelson and Lefebvre, 1995; Dent et al., 2005).

A translocation occurs when a chromosome breaks and the fragmented pieces re-attach to a different broken chromosome. Creighton and McClintock (1931) took advantage of a maize line that was heterozygous for a translocation between chromosomes 8 and 9 and carried a cytologically visible knob. Screening recombinant progeny provided irrevocable evidence that recombination involved physical exchange of DNA. Comparable results were also observed in *Drosophila* by Stern (1934). Many species of evening primrose carry multiple reciprocal translocations and form multi-chromosomal meiotic rings involving all 14 chromosomes (Cleland, 1972). Evening primrose plants produce gametes with normal chromosomes and balanced translocation chromosomes. Chromosome rearrangements generated by gene editing have been reported in human cell lines (Kraft et al., 2015; Adikusuma et al., 2018; Kosicki et al., 2018; Cullot et al., 2019; Rayner et al., 2019). This should not be unexpected, as the broken ends of double strand breaks (DSBs) generated in a CRISPR-Cas9 experiment at target sites and off-target sites within a cell may fuse to form chromosomal translocations or inversions. CRISPR-Cas9 gene-editing techniques have been used to intentionally make chromosomal aberrations in *Arabidopsis* (Rönspies et al., 2022) and in mammalian cells. The engineering of chromosomal changes has produced large deletions and insertions (Eleveld et al., 2021) as well as inversions and translocations (Jiang et al., 2016; Vanoli et al., 2017; Jeong et al., 2019).

Two of three strains we obtained by targeting the *PF23* locus show meiotic viability patterns that are consistent with translocations. As observed in *S. cerevisiae* (Loidl et al., 1998), the frequency of inviability changes with the location of the centromere relative to the breakpoint. The proximity of the chromosome 3 breakpoint to the centromere may be associated with the increased number of tetrads with one viable progeny. The generation of translocations seems to be specific to CRISPR-mediated mutagenesis in *Chlamydomonas*. Previously, we analyzed 20 insertional strains by tetrad analysis and failed to find patterns of meiotic lethality suggestive of chromosomal aberrations (Lin et al., 2018). We do not know the frequency of chromosome aberrations associated with gene-editing events in *Chlamydomonas*, as most researchers do not perform meiotic crosses with the edited strains. It will be important to determine how frequently chromosome aberrations are generated. Monitoring and minimizing cells with chromosome aberrations will be crucial for future applications of genome editing in *Chlamydomonas*.

## Methods

### *Chlamydomonas* strains, media, and growth conditions

*C. reinhardtii* strains (Supplemental Table 16) were maintained on Sager and Granick medium supplemented with acetate (Sager and Granick, 1953). Strains CC-5908 and CC-5909 *ac17*; *atg17*::*aphVIII* were generated by a cross of strain 6B10 containing the *aphVIII* insertional construct in the 3′ UTR of the *ATG17* gene (Lin et al., 2018) and strain *nit2*; *ac17*; *ery1.* It was originally published as *ATG11*. CC-4321 containing the *ac17* mutation was obtained from the *Chlamydomonas* Resource Center.

### Isolation of autolysin

Mating-type plus and minus strains (CC-124 and CC-125) were inoculated with 10^7^ cells and grown for 3 days on Sager and Granick medium (Sager and Granick, 1953) under constant illumination at 25°C and left at room temperature for 2 days (Dutcher, 1995). To promote the transition from vegetative to gametic cells, each plate was transferred to 10 ml of liquid M-N/5 medium and incubated at 25°C with constant illumination and stirring for 3 h. Readiness for mating was determined by phase contrast microscopy for the presence of clusters of cells using 10-μl samples of each parent. If >80% of the cells appeared in clusters, the two strains were mixed and incubated again at 25°C under constant illumination with no stirring for 30–45 min. The mating mixture was checked by phase contrast microscopy with a 40× objective for the presence of quadriciliate cells to evaluate the efficiency of mating. After 30 min, >90% of cells had mated. The mixture was centrifuged at 800 *g* for 5 min at 4°C, and the clear supernatant containing the crude autolysin extract was decanted into a flask on ice. The crude autolysin extract was filter sterilized through a 0.45-μm filter and frozen at −80°C in 15-ml aliquots. Efficacy of autolysin was determined by incubating 1 × 10^7^ gametic cells in thawed autolysin for 30 min with a range of volumes. Then 18 μl of cells from each volume of autolysin were mixed with 2 μl of 2% NP40 and examined by phase contrast microscopy. Cells that have lost the cell wall lyse. The smallest volume of autolysin in which there is complete cell lysis was used to determine the optimal volume to use for DNA extraction and isolation (typically around 1 ml per 10^7^ cells).

### High-molecular-weight genomic DNA extraction

Cells for DNA isolation were grown as described above for autolysin production. The total number of cells was determined using an Eppendorf BioSpectrometer at an absorbance of 680 nm using a standard curve and centrifuged at 5000 *g* for 2 min. The pellet was resuspended in a volume of autolysin as determined above. Treated cells were centrifuged at 5000 *g* for 2 min. The pellet was transferred into a 1.5-ml tube. If the number of cells in the pellet exceeded 2.5 × 10^8^ cells, the pellet was divided into additional tubes for the remainder of the isolation. Samples were centrifuged at 5000 *g* for 2 min to remove excess supernatant. The cell pellet was homogenized using a Monarch single-use microtube pestle for 30 s. The pestle was washed with 180–360 μl of HMW gDNA Tissue Lysis Buffer (Monarch), with larger volumes used for highly viscous samples, and retained in the same tube. The homogenized sample was immediately resuspended, transferred to a 2-ml tube containing 40 μl of 20 mg/ml proteinase K (QIAGEN), and incubated on an Eppendorf ThermoMixer C at 900 rpm for 30 min at 56°C. After proteinase K incubation, 10 μl of 100 mg/μl RNase A (QIAGEN) was added to the sample and incubated at 900 rpm for an additional 10 min at room temperature. All steps following sample lysis were performed with the MagAttract HMW DNA Kit (QIAGEN) following the manufacturer’s protocol. To facilitate complete homogenization of the magnetic bead pellet, elution parameters were changed to a 30-min incubation at 900 rpm at 60°C, resulting in a significant increase in DNA yield and molecule sizes. DNA sample concentrations were quantified using an Invitrogen Qubit 4 Fluorometer (Thermo Fisher Scientific), and molecule size distributions were estimated using a FEMTO Pulse system (Agilent). Samples were further cleaned and concentrated using AMPure XP SPRI paramagnetic beads (Beckman Coulter) at a DNA:bead volume ratio of 1:1.8, followed by two washes using freshly prepared 70% ethanol and resuspension in nuclease-free water (QIAGEN). Low-concentration samples were pooled together as necessary during SPRI bead clean-up to increase the concentration. For freeze and thaw optimization tests, DNA was stored at −20°C and thawed at room temperature for 1 h before being returned to −20°C.

### PacBio HiFi sequencing

A 16-μg sample in 200 μl of nuclease-free water was sent to McDonnell Genome Institute (Washington University in Saint Louis) for library preparation and sequencing according to the standard protocol of PacBio (Pacific Bioscience, www.pacb.com/). A HiFi library was generated by (1) genomic DNA shearing, (2) DNA damage repair and end repair, (3) ligation of SMRTbell hairpin adapters, (4) isolation of target fragment sizes using SageELF (Sage Science) fractions 1–18 kb, and (5) DNA polymerase binding. The resulting SMRTbell library was sequenced on one SMRT cell 8M on a Sequel II instrument. Consensus reads (CCS reads) were generated using the CCS algorithm (https://github.com/PacificBiosciences/ccs), generating 18.2 Gb of CCS ≥ Q20 data.

### ONT sequencing

Samples with concentrations between 50 and 150 ng/μl were subjected to size selection for DNA molecules >25 kb using the Circulomics Short Read Eliminator Kit (Pacific Biosciences). Sequencing libraries were prepared using the standard protocol of the ONT Ligation Sequencing Kit (SQK-LSK110) (ONT, https://nanoporetech.com/) with the following modifications. DNA repair and end prep were performed in the 1.5-ml tube used for storage with incubation steps in a thermal block to reduce the amount of pipetting, and the 20°C incubation time was increased to 30 min. During subsequent AMPure XP bead clean-up, incubation of the bead pellet was increased to 10 min with occasional flicking of the tube. Incubation of the adapter ligation reaction was increased to 20 min. For the final AMPure XP bead clean-up step, the sample was incubated for 10 min before washing the pellet. R9.4.1 flow cells were loaded with libraries ranging between 182 and 1484 ng. Once <10% of pores were actively sequencing, the flow cell was washed using the ONT Flow Cell Wash Kit (EXP-WSH004) and reloaded with a new library. ONT Flongles were primed with 100 μl of flush buffer (prepared by mixing 117 μl of flush buffer with 3 μl of flush tether) and loaded with 29 μl of library that was prepared by mixing 5 μl of DNA, 13.5 μl of sequencing buffer, and 11 μl of loading beads.

### Genome assembly, curation, and annotation

HiFi CCS reads were assembled using both Canu (v2.0) (parameters: genomeSize = 120m useGrid = True -pacbio-hifi) (Koren et al., 2017; Nurk et al., 2020) and hifiasm (v0.13) (Cheng et al., 2021) with duplication purging disabled. HiFi assemblies of varying coverages were obtained by adjusting the value of the “readSamplingCoverage” argument for Canu or by randomly subsampling reads using fastqStatsAndSubsample (http://hgdownload.cse.ucsc.edu/admin/exe/) and assembling with hifiasm. Synteny of each assembly’s contigs to CC-1690 (O’Donnell et al., 2020) was determined using NUCmer (v3.1) (Kurtz et al., 2004) and visualized with Dot (https://github.com/MariaNattestad/dot) by processing the .delta file using the DotPrep.py Python script. The assembly with the highest synteny to CC-1690 was then scaffolded using the alignment, with gaps between contigs temporarily filled by adding 60 “N” nucleotides between adjacent contigs. Organellar contigs were identified by BLAST using NCBI reference sequences NC_005353.1 and NC_001638.1 for the chloroplast and mitochondrial genomes, respectively. Contigs with large proportions (>90%) aligning to sequences from bacterial or fungal species using the BLASTn suite against the non-redundant nucleotide collection were flagged as contaminants and removed from the draft assembly. Redundant contigs with repetitive elements spanning the entire length were identified by alignment of the remaining nuclear contigs to the chromosome scaffolds and grouped according to sequence similarity based on multiple sequence alignment using the Clustal Omega (v2.1) Web interface (Madeira et al., 2019) with default parameters. ONT FAST5 files were base called with Guppy (v3.6.1) (parameters: -x "cuda:0" -c dna_r9.4.1_450bps_hac.cfg) (https://nanoporetech.com/) enabled for GPUs using an NVIDIA Tesla V100, and per-base 5-methyl-cytocine frequencies were measured using Nanopolish (v0.13.3) (Simpson et al., 2017). Base-called ONT reads were assembled with NECAT (v0.0.1) (Chen et al., 2021a), Flye (v2.9) (Kolmogorov et al., 2019), and Canu (v2.0) using an estimated genome size of 120 Mb for each assembler. We explored several strategies to fill assembly gaps, taking advantage of the strengths of each sequence type and assembler. Hybrid HiFi-ONT assemblies were generated using QuickMerge (v0.3) (parameters: -l 1000000 -ml 20000) (Chakraborty et al., 2016; Solares et al., 2018). Gap filling of the hifiasm draft assembly was performed with TGS-GapCloser (v1.1.1) (Xu et al., 2020) using both HiFi CCS reads (MINIMAP2_PARAM at line 313 of the main pipeline script was changed from "-x ava-pb" to "-x asm20"; parameters: --ne --tgstype pb) and ONT reads (parameters: --ne), which were trimmed and corrected with Canu. Sequence polishing was performed using RaCon (v1.4.22) (Vaser et al., 2017) with HiFi (parameters: -u --no-trimming -t 8) and ONT (parameters: -m 8 -x -6 -g -8 -w 500 -u -t 8) reads in various combinations. The resulting genome assemblies were assessed using the following metrics: (1) QV (quality) score based on k-mer comparison between the assembly and whole-genome sequencing reads from CC-5816 HiFi and Illumina paired-end reads of parental strains CC-125 (SRR1259171) and CC-124 (SRR1259170) (Lin et al., 2013) using Merqury (v1.3) (Rhie et al., 2020); (2) number of near-universal single-copy orthologs using BUSCO (v5.1.3) (Manni et al., 2021); and (3) the sum length of bases from low-coverage regions, which we define here as regions greater than 5 bp in length covered by five or fewer reads from both HiFi and ONT alignments using BEDTools (v2.27.1) (Quinlan and Hall, 2010). The assembly with the highest QV and BUSCO scores was selected for manual curation. Low-coverage regions were manually inspected using the Integrative Genomics Viewer (IGV) (v2.9.0) (Thorvaldsdóttir et al., 2013). Comparisons with any overlapping HiFi and ONT reads and several of the other ONT and hybrid assemblies were used to determine the likely correction needed to improve sequence quality. Correction strategies included deletion or insertion of sequence, replacing a stretch of sequence with that from another assembly or HiFi read, and re-consensus of targeted regions from HiFi alignments using RaCon. Improvements in QV score, BUSCO score, and read alignments were used to assess whether or not an alteration was retained in the final assembly. Sequences were manipulated using SeqKit (v0.10.1) (Shen et al., 2016). Iso-Seq transcript consensus models were generated using IsoSeq v3 from SMRT Link release 9.0.0 (https://github.com/PacificBiosciences/IsoSeq). Iso-Seq models were merged using the script chain_samples.py from cDNA_Cupcake v28 (https://github.com/Magdoll/cDNA_Cupcake) and classified based on CC-503 v5.7 annotations (Gallaher et al., 2021) using SQANTI3 (https://github.com/ConesaLab/SQANTI3) (Tardaguila et al., 2018).

### Assessment of genome assemblies

Genome statistics were obtained using calN50 (https://github.com/lh3/calN50) and faCount (http://hgdownload.cse.ucsc.edu/admin/exe/). Genome completion was estimated by BUSCO in genome mode with the chlorophyta_odb10 lineage dataset using both MetaEuk (v4) (Levy Karin et al., 2020) and Augustus (v3.4.0) (Stanke et al., 2008) gene predictors. K-mer databases were generated from CC-5816 HiFi CCS reads and whole-genome paired-end Illumina sequencing reads of strains CC-124 and CC-125 using meryl (v1.3) (parameters: k = 18) (Miller et al., 2008). Merqury (v1.3) (Rhie et al., 2020) was used for k-mer completeness analysis and determination of parental haplotypes across chromosomes. Minimap2 (v2.21) (Li, 2018, 2021) was used to align HiFi reads (parameters: -t 8 -ax map-hifi), ONT reads (parameters: -t 8 -ax map-ont -L), contigs from other assemblies (parameters: -t 8 -I 8G -a --eqx -x asm20 -s 5000), and full-length transcripts (parameters: -t 8 -ax splice). BWA-MEM (v0.7.17) (Li and Durbin, 2009) was used for alignment of Illumina short reads. GMAP-GSNAP (v2019-02-15) (Wu and Watanabe, 2005; Wu and Nacu, 2010) was used for alignment of RNA-seq reads (gsnap parameters: -N 1 -A sam -t 8). pbmm2 (v1.2.0) (https://github.com/PacificBiosciences/pbmm2) was used for alignment of Iso-Seq reads (parameters: --preset ISOSEQ --sort --unmapped --log-level INFO).

### Analysis of rDNA, centromeres, organellar insertions, and repetitive sequences

BLAST+ (v 2.11.0) (BLASTn parameters: -task “megablast”) (Camacho et al., 2009) was used to identify rDNA sequences from the following NCBI accessions: NC_005353, NC_001638, X02708, X02706, X02707, KR092109, and AF183463. Clusters of reverse transcriptase-like genes were used to identify centromeres (Lin et al., 2018). The StainedGlass (v0.4) (Vollger et al., 2022) pipeline was used to examine sequence similarity of rDNA and centromere sequences. Repetitive elements were identified using RepeatMasker (v4.0.5) (parameters: -species “Chlamydomonas reinhardtii” -gff) (Smit et al., 2013) and Tandem Repeats Finder (v4.09) (parameters: 2 7 7 80 10 100 2000 -d -m) (Benson, 1999). SEDEF (v1.1) (Numanagić et al., 2018) was used for identification of segmental duplications. Sequences randomly generated to test for false-positive hits of NUPTs and NUMTs were created in a browser application from the Maduro lab (http://www.faculty.ucr.edu/∼mmaduro/random.htm).

### Structural variant detection

Strains *pf23-2* and *pf23-3* were sequenced on R9.4.1 ONT flow cells, and the data were processed as described above. *pf23-2* ONT reads were aligned to CC-5816. Variants were called using Sniffles2 (v2.0.2) (parameters: --max-splits-kb 1 --minsupport 3 --output-rnames –reference ‘CC-5816.fasta’ --tandem-repeats ‘CC-5816_tandemrepeatsfinder2.7.7.80.10.100.2000.bed’) (Sedlazeck et al., 2018) and viewed using IGV and Ribbon (v1.1) (Nattestad et al., 2021). A genome assembly of *pf23-2* using Flye (parameters: --nano-raw --genome-size 120m) was used as a second point of reference for detection of structural variants. Putative translocation partners in *pf23-2* and *pf23-3* were verified through pseudolinkage of molecular PCR markers across the predicted breakpoint (Lin et al., 2018).

### Genome editing

CRISPR genome editing was used to perform insertional mutagenesis to generate null alleles of *PF23*. CRISPRDirect online software (Naito et al., 2015) (https://crispr.dbcls.jp/) was used to select a suitable gRNA sequence in exon 1 of the *PF23* gene. The gRNA with tracrRNA sequence was synthesized by Integrated DNA Technologies (IDT) (cggtttgcccgccatgccgc**TGG**AUGCCGCGUUUUAGAGCUAGAAAUAGCCUUGAAAAAGUGGUCCGUUAUCAAGCACCGAGUCGGUGCUUUU). To promote homologous recombination, a donor DNA sequence was constructed with 50-bp homology arms that matched the regions flanking the *PF23* CRISPR cut site. The insertional construct contained the *aphVII* gene, which confers hygromycin resistance, flanked by left (ACTATTACGAGCCTCCCGACGACTGCTTGACTTCGGTTTGCCCGCC) and right (TACTTCAATTGCCACATCCGTCTCCGTCCATGTATACTTTGGGGCCAGCGGCAT) *PF23* homology arms. Touchdown PCR using Thermo Scientific Phusion polymerase (denaturation 98°C, 10 s; annealing 60°C, 10 s; extension 72°C, 1 min [5×]; denaturation 98°C, 10 s; annealing 72°C, 10 s; extension 72°C, 1 min [30×]) was used to attach the homology arms to the *aphVII* gene. RNPs were assembled using 1.5 μl of spCas9 (IDT, 62 μM stock), 1.5 μl of sgRNA (100 μM stock), and 7 μl of water and incubated for 30 min at room temperature.

Cells for transformation (strain CC-5908 or CC-5909) were grown in 50 ml of liquid TAP medium on a stir plate for 2 days in continuous light at 25°C. Cell density was assessed using a spectrophotometer; cells were then resuspended to a final concentration of 1 × 10^8^ cells/ml using Max Efficiency Transformation Reagent for Algae (Thermo Fisher). Forty microliters of cells, 5 μl of assembled RNPs, and 300 ng of the insertional construct were added to a 2-mm cuvette. Transformation was performed using a Nepa Gene electroporator using voltage parameters 250 V poring pulse with 8-ms pulse length, 50-ms pulse interval, and 40% decay rates (Yamano et al., 2013). After electroporation, cells were incubated in the cuvettes at 25°C for 5 min and then transferred to 8 ml of TAP medium with 150 mM D-mannitol. Cells were allowed to recover by rocking overnight in the dark at 25°C. The next day, cells were spun down at 1000 *g* for 5 min, resuspended in 1 ml of TAP medium, and spread onto ¾ TAP + hygromycin plates. The TAP medium uses only ¾ of the acetate in standard recipes; this increases the efficacy of the hygromycin. Colonies were picked after a 4- to 5-d incubation in continuous light at 25°C. *PF23*- and *aphVII*-specific primers flanking the insertion site were used to verify the presence of the insertion.

### Tetrad analysis

Matings were performed as described previously (Dutcher, 1995). Because the cilia are short in the *pf23* strains, dibutyryl cyclic AMP (cAMP) and 3-isobutyl --methylxanthine (IBMX) were added for 30 min before mixing the parents (Pasquale and Goodenough, 1987). To increase the number of tetrads with four and not eight progeny, the zygotes were kept at 21°C. Strains 3-3 and 1-1 were picked from progeny of a cross of CC-124 and CC-125 to obtain parents that would result in heterozygosity of PCR-based markers on chromosomes 5 and 11 in crosses with *pf23-2*. PCR primers (Supplemental Table 1) were used to score 25 tetrads.

### Immunofluorescence

Cells were grown on Sager and Granick medium for 2 days (Sager and Granick, 1953). They were then resuspended in 500 μl of M-N/5 gametogenic medium for 3 h in light and allowed to rock at room temperature for 3 h. Cells were then treated with 500 μl of autolysin for 40 min, spun down at 10 000 *g* for 1 min, and resuspended in 500 μl of microtubule stabilization buffer (MTSB) (Pasternak et al., 2015). The cells were then placed onto 1.5 mm × 8 mm × 8 mm coverslips that were pretreated with L-polylysine (Sigma) for 10 min, rinsed, and then air dried. After allowing the cells to settle in the dark for 1 min, cells were aspirated, and the slides were washed once with PBS. Coverslips were then immersed in ice-cold methanol for 5 min twice to allow fixation. Coverslips were allowed to air dry and then rehydrated with PBS for 10 min followed by blocking for 1 h with 100% blocking buffer (0.05% BSA and 1% fish gelatin in PBS + 0.02% Tween 20). Coverslips were then incubated with the primary antibody mouse acetylated α-tubulin at a 1:1000 dilution (Sigma/Millipore) in 20% blocking buffer for 1 h at room temperature (or 4°C overnight), then washed three times for 5 min with 20% blocking buffer. Alexa Flour 488 goat anti-rat-IgG antibody (Invitrogen API83P, lot number 2915317, 1:500 dilution) was added 1:1000 for 1 h, washed three times for 5 min with 20% blocking buffer, and then mounted and sealed. Images were acquired at 63× magnification using a Zeiss Axioplan spinning disk confocal microscope with PerkinElmer software.

### Immunoblotting

Immunoblotting was performed using whole-cell extracts of CC-5908 and *pf23* strains (Lin et al., 2015; Hunter et al., 2018). The primary antibody rabbit anti-PF23 CT299 was used at a 1:1000 dilution (Patel-King et al., 2019), and NAB1 rabbit antibody was used at a 1:10 000 dilution (Agrisera, lot #1311) overnight. The membrane was visualized using anti-rabbit horseradish peroxidase (HRP) (Sigma A6154, 1:5000 dilution) conjugated secondary antibody.

## Funding

This work was supported by a grant (R35GM131909) to S.K.D. and a grant (R00MH117165) to T.N.T. G.M.P. was supported by The Bayer Excellence Fund for Graduate Fellowships in Life Sciences.

## Author contributions

Z.L.P., G.M.P., and S.K.D. conducted the experiments. Z.L.P., G.M.P., T.N.T., and S.K.D. performed analysis. Z.L.P., G.M.P., and S.K.D. wrote the manuscript.

## Accession numbers

The CC-5816 *C. reinhardtii* genome assembly is available at NCBI under GenBank assembly accession number GCA_026108075.1 and can be viewed at the University of California, Santa Cruz (UCSC) genome browser (https://genome.ucsc.edu/h/GCA_026108075.1). The raw sequencing reads generated in this study are deposited under NCBI BioProject PRJNA840945.

## Supplemental information

Document S2. Supplemental Tables 1–12 and 17

Document S3. Article plus supplemental information
