# Supplementary material for: A gap-free genome assembly of Chlamydomonas reinhardtii and detection of translocations induced by CRISPR-mediated mutagenesis
Source: Plant Commun. 2022 Nov 17;4(2):100493. doi: 10.1016/j.xplc.2022.100493 (PMC10030371; doi:10.1016/j.xplc.2022.100493)
Supplement: Document S1. Supplemental Figures 1–7 and Supplemental Tables 13–16 [file mmc1.pdf]

**Plant Communications, Volume *4***

## Supplemental information

# A gap-free genome assembly of *Chlamydomonas reinhardtii* and detection of translocations induced by CRISPR-mediated mutagenesis

**Zachary L. Payne, Gervette M. Penny, Tychele N. Turner, and Susan K. Dutcher**

# A gap-free genome assembly of *Chlamydomonas reinhardtii* and detection of translocations induced by CRISPR-mediated mutagenesis

Zachary L. Payne, Gervette M. Penny, Tychele N. Turner, and Susan K. Dutcher*

Department of Genetics, Washington University School of Medicine, Saint Louis, MO 63110, USA

***Contact: Susan K. Dutcher (dutcher@wustl.edu)**

## Supplemental Figures and Tables

Supplemental Figure S1. CC-503 v5 assembly of *Chlamydomonas reinhardtii* contains chromosomal rearrangements and mis-assemblies with respect to wild-type strain CC-5816.

Supplemental Figure S2. Optimizing extraction of HMW DNA from *Chlamydomonas reinhardtii*.

Supplemental Figure S3. PacBio HiFi and Nanopore raw sequencing results.

Supplemental Figure S4. Comparison of raw assemblies generated by Hifiasm and Canu.

Supplemental Figure S5. Identification of CRISPR/Cas9-induced translocation breakpoints in *pf23-2* using the CC-5816 vs CC-503 v5 genome assemblies.

Supplemental Figure S6 Translocation in *pf23-3* was induced by site-directed CRISPR/Cas9 mutagenesis.

Supplemental Figure S7. FAP221 and FAP360 gene model correction.

Supplemental Table S13: Primers used in studies

Supplemental Table S14: Expected amplicon sizes for primers listed in Supplemental Table S13 strains in Figure 6

Supplemental Table S15: Behavior of chromosomes in meiotic progeny in tetrads from heterozygous translocation crosses

Supplemental Table S16: Strains used in this study

## Supplemental Tables (Excel files included as Supplemental Information):

Supplemental Table S1: Hifiasm draft assembly contigs

Supplemental Table S2: Evaluation of all assemblies generated using different algorithms

Supplemental Table S3: Assembly gaps filled by TGS-GapCloser

Supplemental Table S4: Manual fixes of the assembly

Supplemental Table S5: Low coverage regions in the final assembly

Supplemental Table S6: Repeats identified by RepeatMasker and Tandem Repeats Finder

Supplemental Table S7: Centromere length and methylation level

Supplemental Table S8: Compilation of IsoSeq consensus models

Supplemental Table S9: IsoSeq transcripts in the centromere

Supplemental Table S10: Analysis of centromeric gene clusters

Supplemental Table S11: Location, length, and coverage of organellar DNA insertions into the nuclear genome

Supplemental Table S12: BLAST alignments of organellar insertions

Supplemental Table S17: Nuclear coding sequence found linked to a plastid Hifiasm contig

## Supplemental Figures and Tables

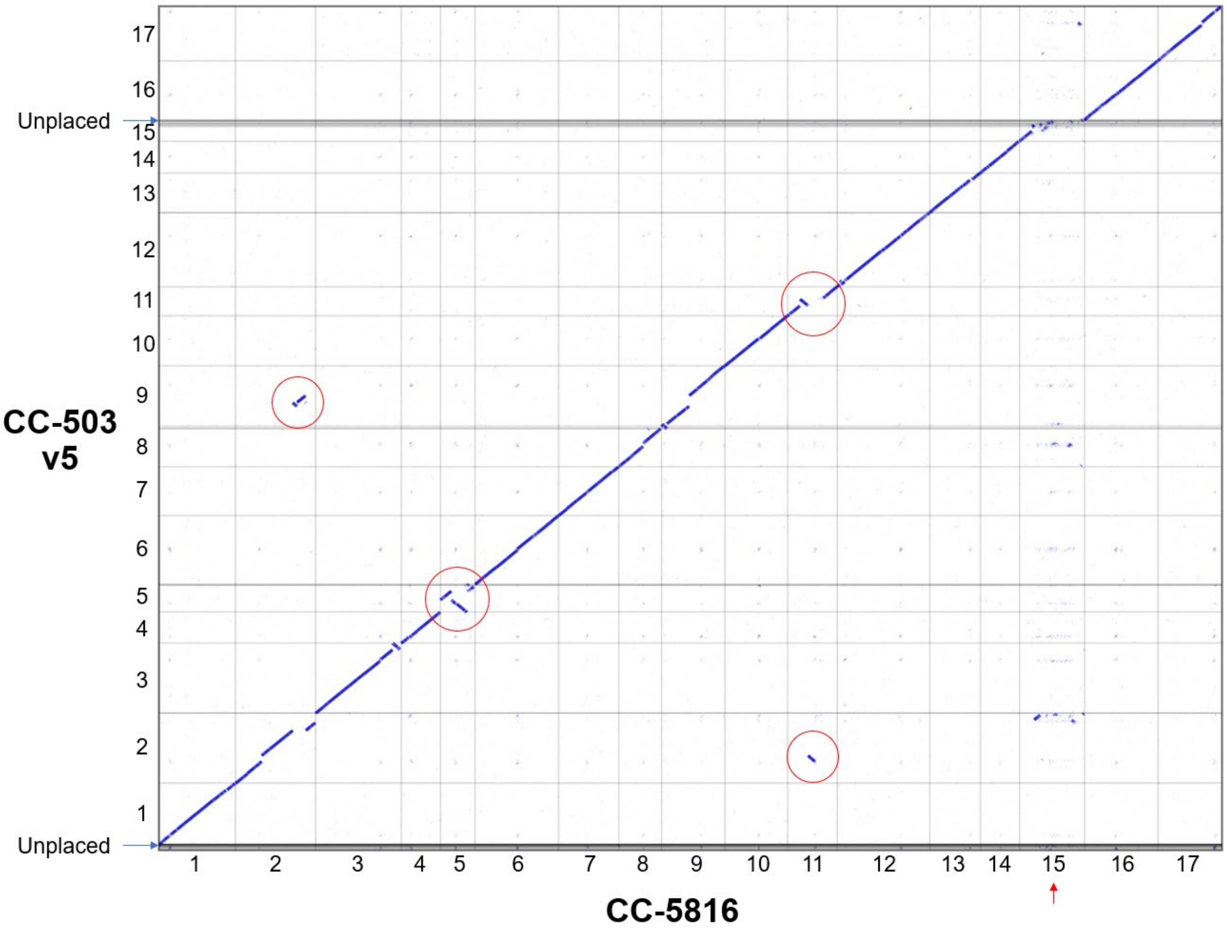


### Supplemental Figure S1. CC-503 v5 assembly of *Chlamydomonas reinhardtii* contains chromosomal rearrangements and mis-assemblies with respect to wild-type strain CC-5816.

The dotplot shows comparison of the CC-5816 assembly generated in this study (x-axis) to the CC-503 v5 assembly generated at the U.S. Department of Energy Joint Genome Institute (y-axis). Aligned sequence is indicated by blue line. Chromosomes are labeled 1-17 for each assembly. Large numbers of unplaced scaffolds appear as thick or densely aggregated horizontal bars. Two obvious examples are labeled as unplaced in the figure. Red circles highlight multiple regions where there are either mis-assemblies or chromosomal rearrangements in CC-503 compared to CC-5816. The red arrow points to chromosome 15, which is particularly fragmented in CC-503.

**A**

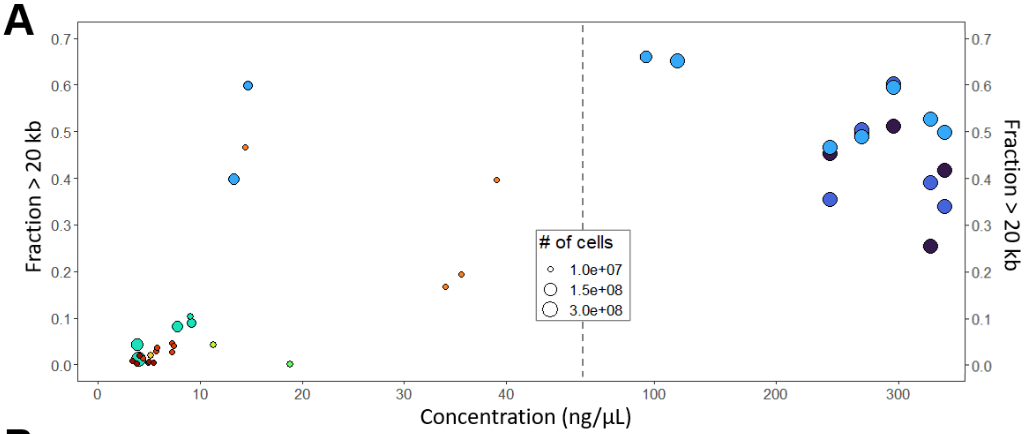


**B**

| Input cells | Autolysin treated | Homogenized | Lysis buffer | ThermoMixer Elution | Sample storage |
|---|---|---|---|---|---|
| Frozen | No | No | SDS | 25°C, 1400 RPM, 3 min | -20 °C |
| Vegetative | No | No | SDS | 25°C, 1400 RPM, 3 min | 4 °C |
| Gametic | Yes | No | SDS | 25°C, 1400 RPM, 3 min | 4 °C |
| Gametic | Yes | Yes | SDS | 25°C, 1400 RPM, 3 min | 4 °C |
| Gametic | Yes | No | Water | 25°C, 1400 RPM, 3 min | 4 °C |
| Gametic | Yes | Yes | Water | 25°C, 1400 RPM, 3 min | 4 °C |
| Gametic | Yes | Yes | EDTA | 25°C, 1400 RPM, 3 min | 4 °C |
| Gametic | Yes | Yes | EDTA | 60°C, 900 RPM, 30 min | 4 °C |
| Gametic | Yes | Yes | EDTA | 60°C, 900 RPM, 30 min | -20 °C (1x freeze/thaw) |
| Gametic | Yes | Yes | EDTA | 60°C, 900 RPM, 30 min | -20 °C (5x freeze/thaw) |

## Supplemental Figure S2. Optimizing extraction of HMW DNA from *Chlamydomonas reinhardtii*.

**(A)** Optimization of DNA isolation was conducted by analyzing samples extracted with several parameters altered. Samples were analyzed using FEMTO pulse gel electrophoresis. The yield from each isolation, shown as sample concentrations, were compared to the fraction of the sample containing DNA molecules >20 kb in length. DNA molecules were calculated using the formula:

$$ug\ DNA \times \frac{pmol}{660\ pg} \times \frac{10^6\ pg}{1\ ug} \times \frac{1}{N} = pmol\ DNA$$

**(B)** Parameters that were altered for each experiment in **(A)**.

A

B

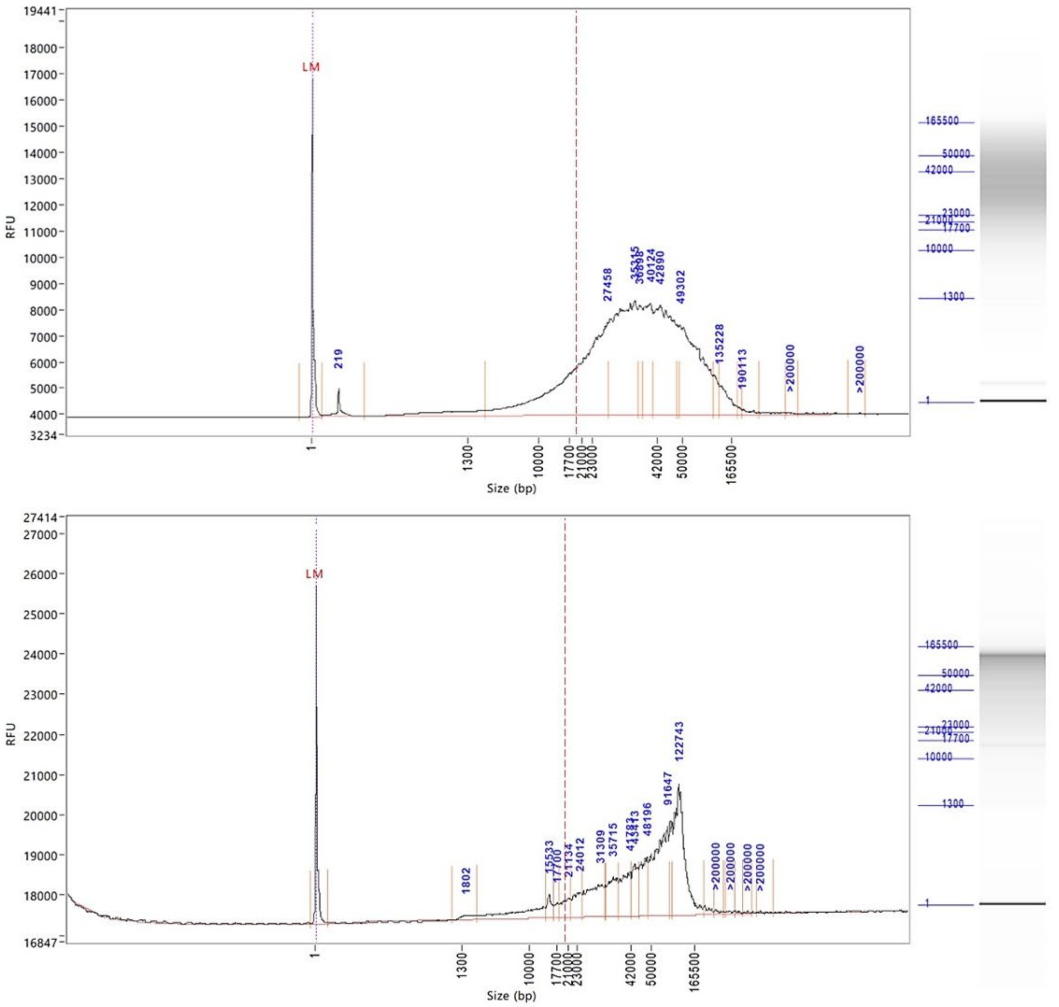


C

| | PacBio HiFi | Nanopore |
|---|---|---|
| **Total bases** | 18,259,845,352 | 6,480,130,808 |
| **Number of reads** | 987,786 | 346,684 |
| **Coverage** | 157x | 51x |
| **Read length N50 (bp)** | 17,883 | 39,357 |
| **Mean read length (bp)** | 18,486 | 18,692 |
| **Longest read (bp)** | 42,677 | 338,467 |
| **Mean Phred quality score** | 27.3 | 11.7 |

D

E

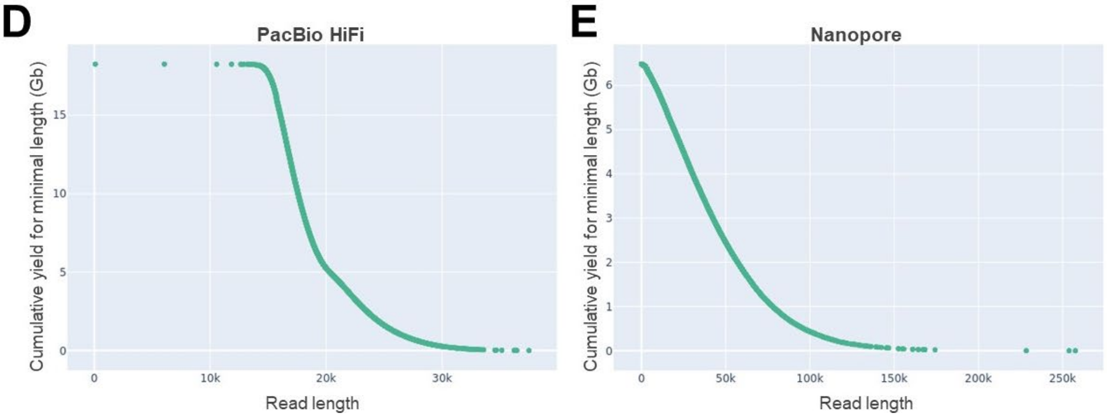

## Supplemental Figure S3. PacBio HiFi and Nanopore raw sequencing results.

**(A and B)** FEMTO pulse traces and the corresponding gel for samples used for **(A)** PacBio HiFi sequence and **(B)** Nanopore sequencing. The red dotted horizontal line indicates separation of DNA molecules in the sample less and greater than 20 kb. **(C)** Table containing general statistics of each read set. **(D)** Contribution of the total yield *y* in Gb from reads size *x* and longer.

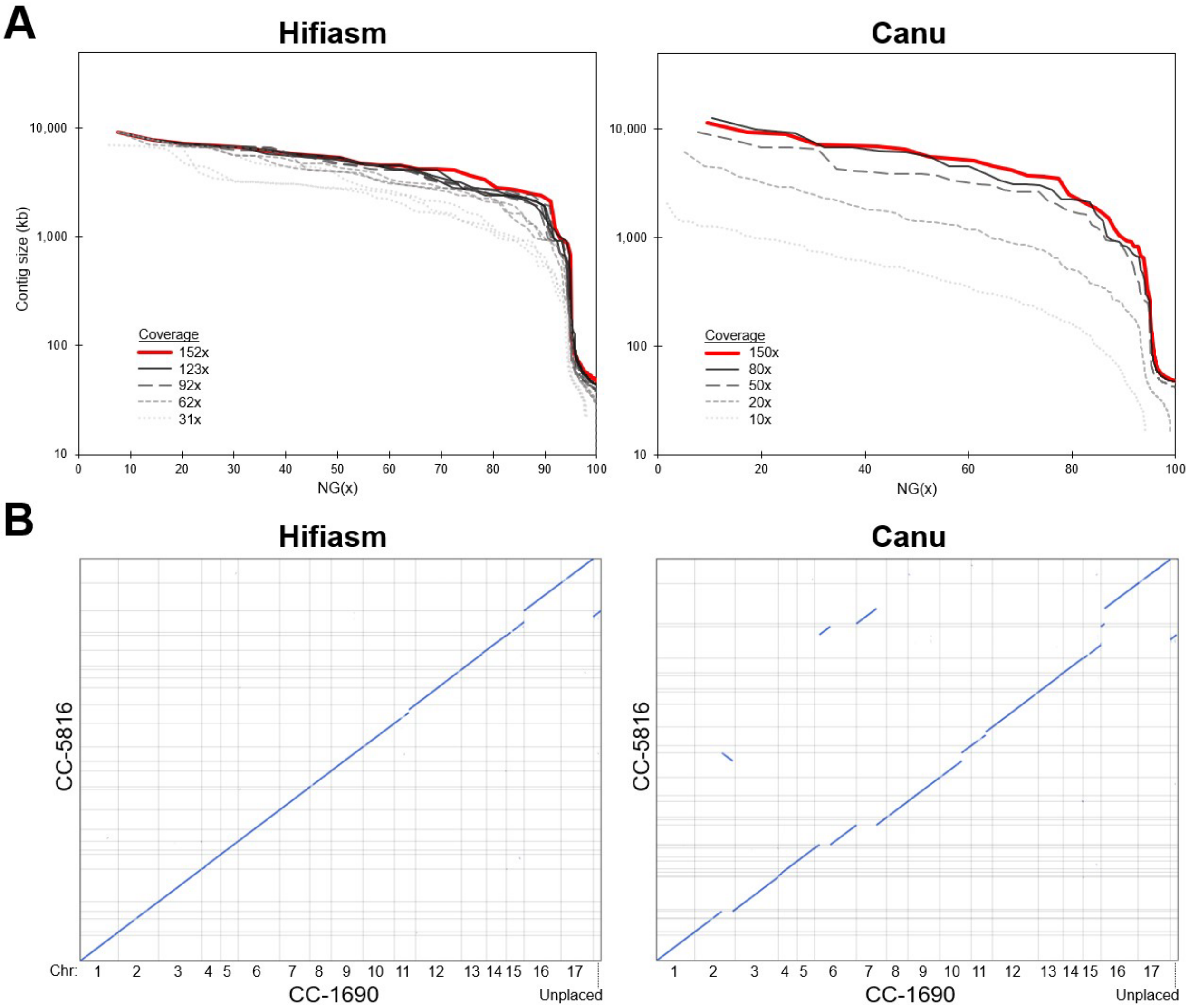


## Supplemental Figure S4. Comparison of raw assemblies generated by Hifiasm and Canu.

**(A)** PacBio HiFi reads were assembled at full coverage and at select downsampled coverages using Hifiasm (3 replicate downsamples per coverage and one assembly at full coverage) and Canu (one assembly per coverage). Each assembly is represented by a curve. The cumulative fraction of the total assembly size *x*, contributed from contigs sizes *y* bp and shorter. **(B)** Dot plots comparing an assembly from strain CC-1690 to CC-5816 assemblies obtained from Hifiasm and Canu using full coverage from PacBio HiFi reads. Two large mis-assemblies can be seen in the Canu assembly for chromosomes 2, 6, 7, 10, 15, and 16, which are not seen in the Hifiasm assembly.

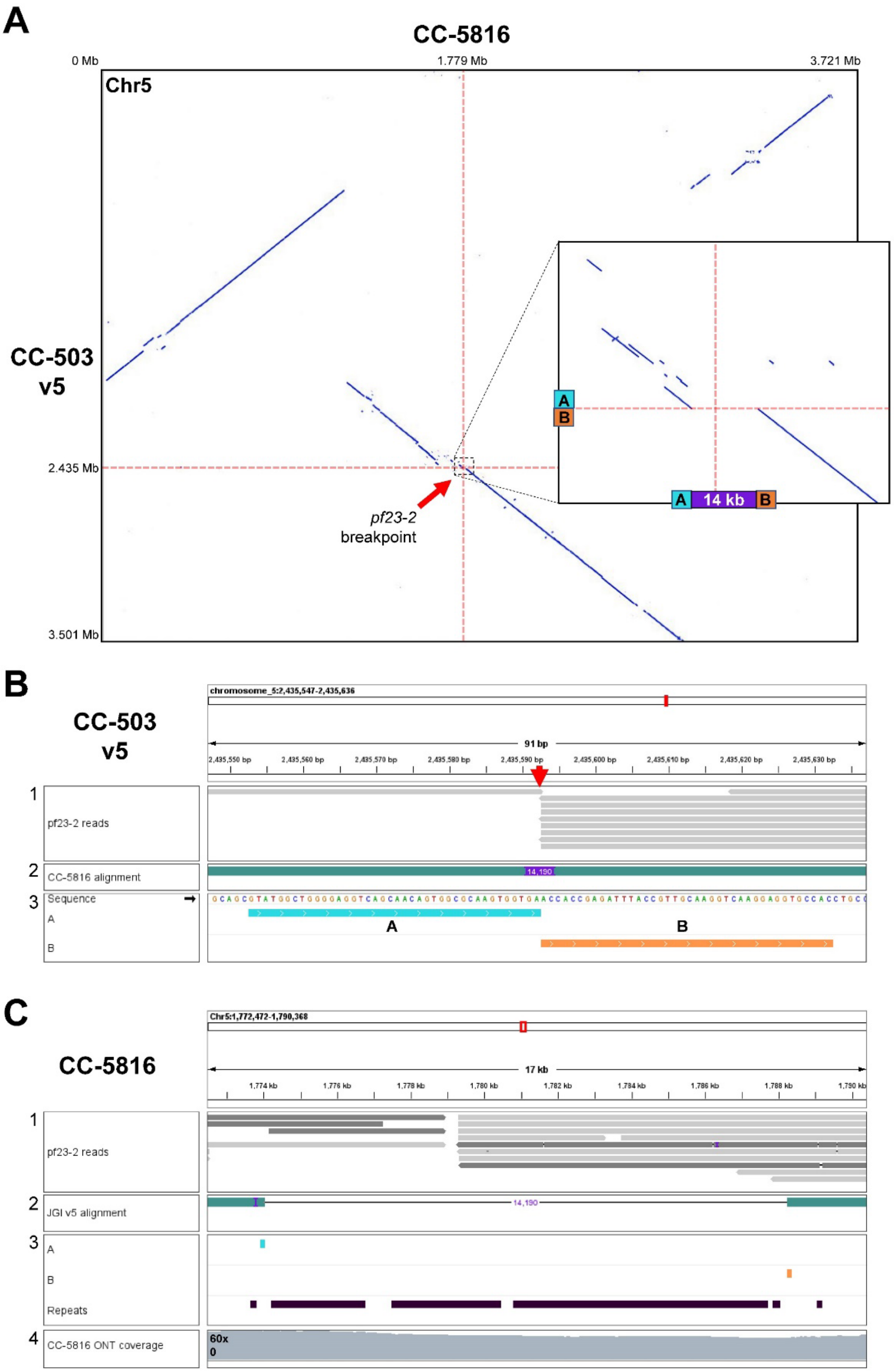
A
CC-5816
0 Mb
1.779 Mb
3.721 Mb
Chr5
CC-503
v5
2.435 Mb
3.501 Mb
pf23-2
breakpoint
A
B
A
14 kb
B
B
CC-503
v5
chromosome_5:2,435,547-2,435,636
91 bp
2,435,550 bp
2,435,560 bp
2,435,570 bp
2,435,580 bp
2,435,590 bp
2,435,600 bp
2,435,610 bp
2,435,620 bp
2,435,630 bp
1
pf23-2 reads
2
CC-5816 alignment
14,190
3
Sequence
A
B
A
B
C
CC-5816
Chr5:1,772,472-1,790,368
17 kb
1,774 kb
1,776 kb
1,778 kb
1,780 kb
1,782 kb
1,784 kb
1,786 kb
1,788 kb
1,790 kb
1
pf23-2 reads
2
JGI v5 alignment
14,190
3
A
B
Repeats
4
CC-5816 ONT coverage
60x
0

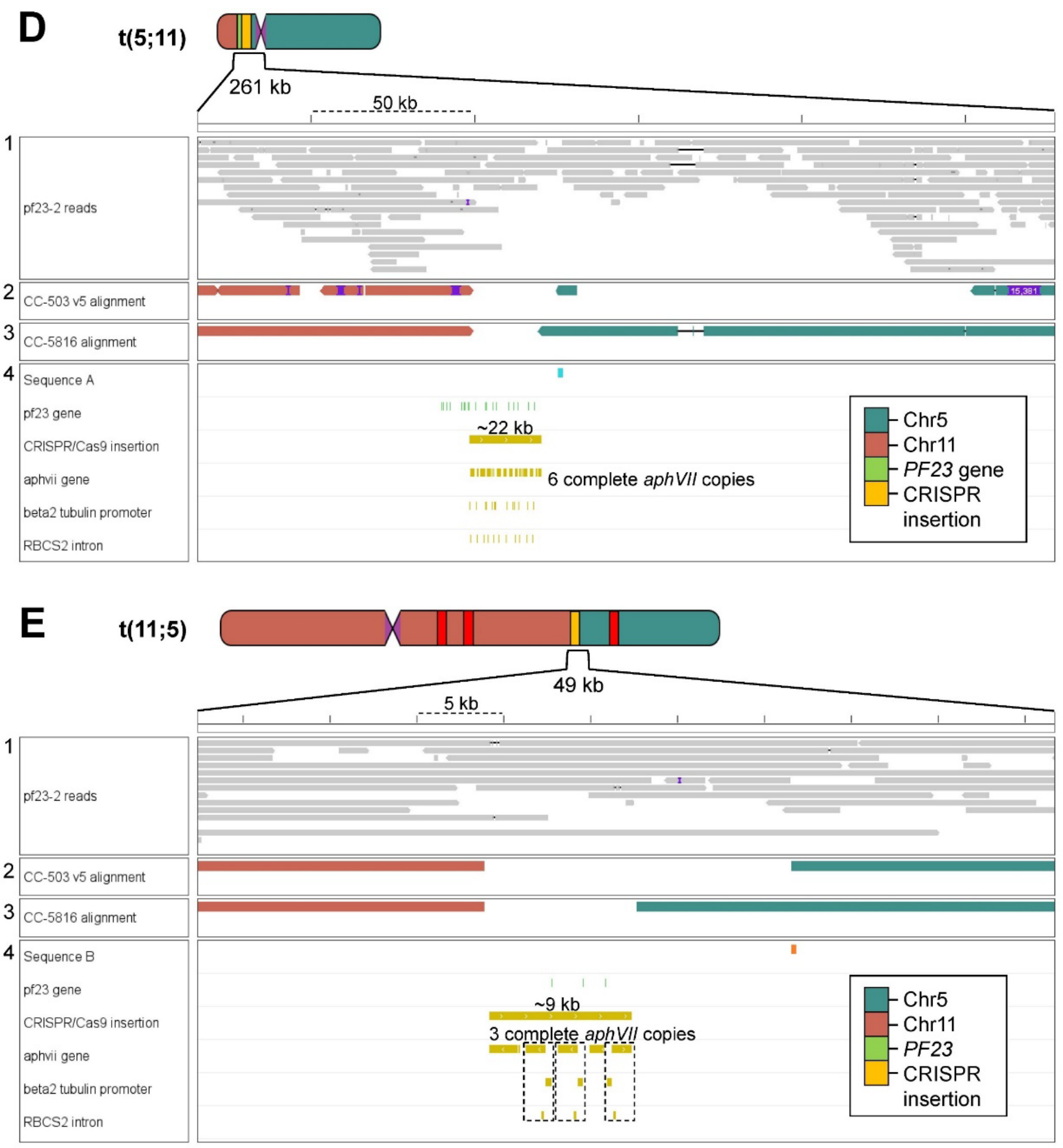


## Supplemental Figure S5. Identification of CRISPR/Cas9-induced translocation breakpoints in *pf23-2* using the CC-5816 vs CC-503 v5 genome assemblies.

**(A)** Dotplot showing alignment between CC-5816 (x-axis) and CC-503 v5 (y-axis) on Chr5. Predicted breakpoint from *pf23-2* on Chr5 was determined using both genome assemblies and denoted with the dotted red line. A zoomed in view of the breakpoint region is denoted by the dotted black box and lines. The *pf23-2* breakpoint determined from CC-503 v5 (horizontal red dotted line) is located precisely where there is a 14 kb deletion with respect to CC-5816 (purple box). The same analysis performed using CC-5816 results in the breakpoint falling within this 14 kb sequence missing from CC-503 v5 (vertical red dotted line). 41 bp of sequence on either side of the breakpoint determined with CC-503 v5 are denoted as A (turquoise) and B (orange) along the y-axis, which were then located in CC-5816, shown again as turquoise and orange boxes along the x-axis. **(B and C)** IGV screenshots of the breakpoint determined by using either CC-503 v5 or CC-5816, respectively. Panel 1 shows aligned *pf23-2* reads, with the breakpoint denoted with a red arrow; light grey-colored reads indicate reads that are visible in both (B) and (C). Panel 2 shows alignments from either (B) CC-5816, or (C) CC-503 v5. Panel 3 displays sequences A (turquoise) and B (orange) which flank the breakpoint identified using CC-503 v5, as

described in (A). **(C)** The bottom of panel 3 displays repetitive sequence for CC-5816, which is particularly abundant within the 14 kb sequence missing in the CC-503 v5 genome where the precise *pf23-2* breakpoint is located. Panel 4 displays 0x to 60x coverage from CC-5816 Nanopore reads within this region. Good coverage across this region indicates it appears to be assembled correctly in CC-5816. **(D and E)** IGV screenshot of the breakpoints and insertions on each the two translocated chromosomes assembled *de novo* with Flye using *pf23-2* Nanopore reads. The chromosome ideograms from Figure 7D are shown above with brackets indicating the approximate region visible in the IGV screenshot below. Reads from *pf23-2* Nanopore sequencing are shown in panel 1. Panels 2 and 3 show alignments from CC-503 v5 and CC-5816, respectively. Chr5 alignments are colored blue, Chr11 alignments are colored brown, insertions within alignments are colored purple, thin lines between colored bars indicate a gap in the alignment, and regions with no colored bars indicates no alignment. The fourth panel displays *pf23* coding sequence in green, and features of the insertion cassette in yellow. Individual components of the insertion cassette, including the *aphVII* gene, beta2 tubulin promoter, and *RBCS2* intron are shown below the CRISPR/Cas9 insertion row, also in yellow. Multiple tandem copies of the insertion cassette are inserted at the breakpoint, some contain intact copies of the *aphVII* gene, and some are only partial sequence. Sequences A (turquoise) and B (orange) described previously are 41bp sequences on either side of the *pf23-2* breakpoint when using CC-503 v5 for analysis. The IGV screenshots show **(D)** a 261 kb region spanning the breakpoint and insertion region on the smaller translocation partner and **(E)** a 49 kb region spanning the breakpoint and insertion region on the larger translocation partner. Precise breakpoints on Chr11 can be determined using both CC-503 v5 and CC-5816 assemblies. However, precise breakpoints from Chr5 can only be determined using CC-5816, demonstrated by the lack of sequence from CC-503 v5 proximal to the insertion sequence.

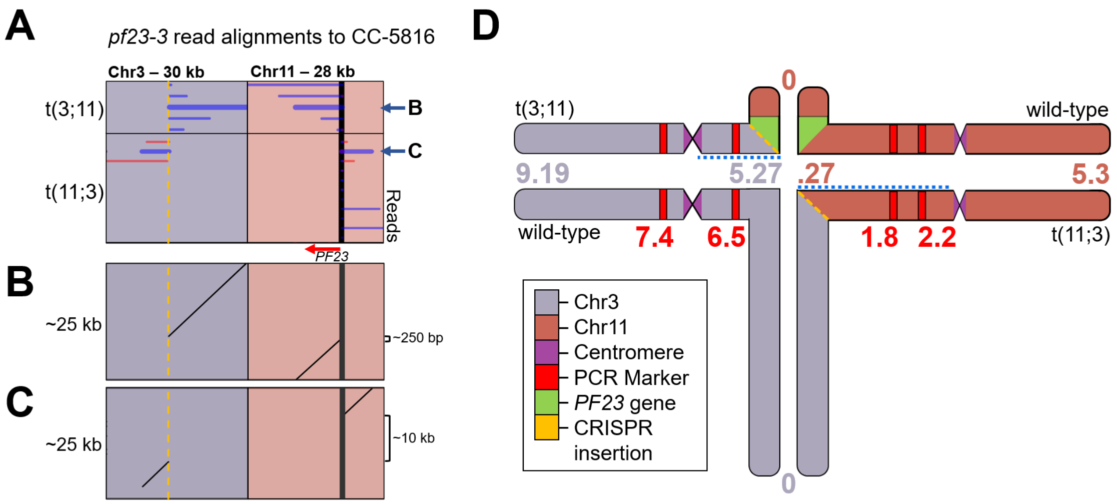


## Supplemental Figure S6 Translocation in *pf23-3* was induced by site-directed CRISPR/Cas9 mutagenesis.

**(A)** Alignments to CC-5816 from *pf23-3* Nanopore reads within a 30kb region on Chr3 (purple) and a 28kb region on Chr11 (brown-red) containing the *pf23* gene locus (dark red box). The approximate CRISPR/Cas9 cut site in exon one is shown as the thick black vertical bar and the breakpoint on Chr3 as the dotted yellow line. Each row corresponds to a single *pf23-3* read with blue and red colors indicating forward and reverse alignments, respectively. Reads are separated by the black line to indicate which translocation partner the reads correspond to. Reads B and C in bold indicated by blue arrows refer to the following panels which show alignments in greater detail.

**(B)** Dotplot of a ~25kb read, bolded read B in panel (A), which spans the ~250 bp insertion on chromosome t(3;11) containing no copies of the *aphVII* gene. **(C)** Dotplot of a ~25kb read, bolded read C in panel (A), which spans the ~10 kb insertion on chromosome t(11;3) containing at least 3 full copies of *aphVII* and several truncated copies. **(D)** Quadrivalent structure of Chr3, Chr11, t(3;11), and t(11;3) predicted to form during meiosis I in a *pf23-3* parent × wild-type cross. Coordinates are in Mb and based on CC-5816 to illustrate approximate location of breakpoints. Alternate segregation of chromosomes containing a single crossover between the breakpoints and centromeres (blue dotted line) results in one-half of progeny being inviable due to deletions in either chromosome. Dotted yellow lines denote the location of insertions induced by CRISPR. Centromeres are colored dark purple, PCR markers used for testing linkage are shown as red bars, the approximate location of the *PF23* gene is colored green.

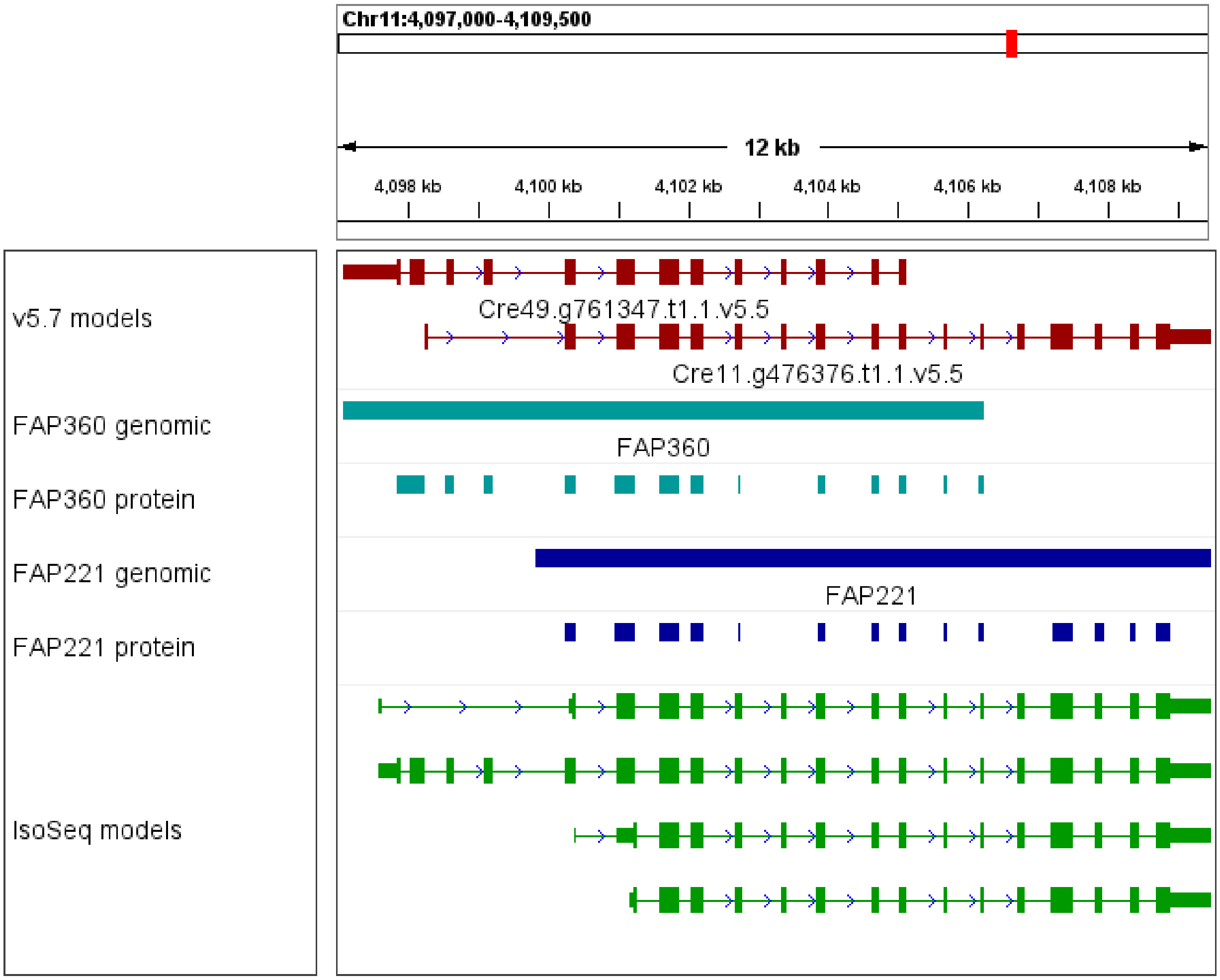


## Supplemental Figure S7. FAP221 and FAP360 gene model correction.

IGV browser screenshot of FAP360 (Cre49.g761347) and FAP221 (Cre11.g476376) gene models aligned to CC-5816 (red), BLAST alignments of genomic and protein sequences of FAP360 and FAP221 (light blue and dark blue, respectively), and IseSeq-derived gene models (green). The evidence presented here suggests these two genes derive from a single locus.

## Supplemental Table S13: Primers used in studies

| Primer name | Forward Sequence | Reverse Sequence |
|---|---|---|
| *PF23-aphVII* junction | TGTAAATGGAGGCGCTCGTTG | GATCGCCAAGCCCTGTTGC |
| Exon 1 CRISPR site | GATCGCCAAGCCCTGTTGC | GTCCAGCGCGAACAGATAGG |
| *PF23* Exon 5 | GTGACGTTCACCCAGCTGG | CTGTTTCCGGAAGGCGCG |
| *PF23* introns 4 and 6 | CATTGGGTACTGGGATGATGGAAC | TGCAGCCGATGACTCCTTATAAGTTT |
| CHR5-1.5 | GCAATGGAACTGGGAGAAGA | CAACAATGCATCCAACACCA |
| CHR11-2.2 | GCAAGTATTGTCAGGCGTTG | TTGGGACTTTGACGGACAG |
| CHR11-1.8 P | AGTCCAAAACGCCTTCATGT | GCCGACCACAGCTACTTTAT |
| CHR3-7.49 P | CAGTGCGAAATCAAGCAGATG | CAGCAAGCTGACAAGTCAAAC |
| CHR3-7.49 C | CTACCTGACAAGGCTGGTTATC | GCGATGCTGCTGCATTTATT |

## Supplemental Table S14: Expected amplicon sizes for primers listed in Supplemental Table S13 for each strain in Figure 6

| | Amplicon size for each strain (bp) | | | |
|---|---|---|---|---|
| **Primer set** | **CC-5908** | ***pf23-2*** | **CC-124** | ***pf23-1*** |
| [A]*PF23-aphVII* junction | no band | 327 | no band | no band |
| [A]Exon 1 CRISPR site | 220 | no band | 220 | 220 |
| [A]Exon 5 | 81 | 81 | 81 | no band |
| [B] *PF23* introns 4 and 6 | 955 | 955 | 955 | 461 |

[A] This study; [B] Yamamoto *et. al.* 2017

## Supplemental Table S15: Behavior of chromosomes in meiotic progeny in tetrads from heterozygous translocation crosses

### Progeny from tetrads with 4 viable progeny with *pf23* alleles

| **Meiotic cross (N = total number of tetrads)** | **Number of tetrads with 4 viable progeny** | **2:2 Segregation of hygromycin resistance** | **Linkage in tetrads with 2:2 segregation of Chr5 2.2Mb and Chr11 1.8 Mb (PD: NPD: T)** | **Tetrads with abnormal segregation of hygromycin resistance** |
|---|---|---|---|---|
| CC-124 x CC-125 N= 28 | 28 | NA | 7:8:13 | None |
| *pf23-2* x 1-1 N=280 | 83 | 75 | 73:1:1 | 8 with 3:1 segregation |
| *pf23-2* x 3-3 N= 344 | 101 | 17 | 16:0:1 | 1 with 3:1 segregation |
| | | | **Linkage in tetrads with 2:2 segregation of Chr3 6.45 (*AC17*) and Chr 11 1.8 Mb (PD: NPD: T)** | **Linkage with 2:2 segregation with Chr3 7.42 Mb** |
| *pf23-3* x 1-1 N=108 | 33 | | 33:0:0 | 24:0:0 |
| *pf23-3* x CC-124 N= 135 | 65 | | 65:0:0 | 32:0:0 |

## Progeny from tetrads with 2 viable progeny

| | Number of tetrads with 2 viable progeny | 1:1 Segregation of hygromycin resistance | Recombination between *pf23-2* and Chr5 2.2 Mb | Recombination between *pf23-2* and Chr5 2.9 Mb | Tetrads with abnormal segregation of hygromycin resistance |
|---|---|---|---|---|---|
| CC-124 x CC-125 | 0 | NA | NA | NA | NA |
| *pf23-2* x 1-1 | 140 | 125 | 14 | 30 | 15 with 2:0 segregation |
| *pf23-2* x 3-3 | 24 | 21 | 4 | 5 | 3 with 2:0 segregation |
| | | | **Recombination between *pf23-3* and *ac17* (6.45 Mb)** | **Recombination between *pf23-3* and Chr3 7.42 Mb** | |
| *ac17 x pf23-1* | | | 14:15:0 | NA | |
| *pf23-3* x 1-1 | 58 | | 58:0:0 | NA | |
| *pf23-3* x CC-124 | 32 | | 32:0:0 | 29:0:3 | |

## Progeny from tetrads with one viable progeny

| | | 1 Swimmer | 1 Pellet | Hygromycin resistant |
|---|---|---|---|---|
| *pf23-3* x CC-124 | 18 | 14 | 4 | 3 |

## Progeny from tetrads with no viable progeny

| | Number of tetrads with no viable progeny |
|---|---|
| CC-124 x CC-125 | 0 |
| *pf23-2* x 1-1 | 57 |
| *pf23-2* x 3-3 | 12 |
| *pf23-3* x 1-1 | 20 |
| *pf23-3* x CC-124 | 22 |

## Progeny from tetrads of *fap70* x wild-type

| | Number of tetrads | | |
|---|---|---|---|
| Mutant | 4 viable: 0 inviable | 2 viable: 2 inviable | 0 viable: 4 inviable |
| *fap70C1* | 4 | 13 | 5 |
| *fap70C4* | 15 | 0 | 0 |
| *fap70D1* | 3 | 14 | 6 |
| *fap70D4* | 3 | 2 | 4 |

## Supplemental Table S16: Strains used in this study

| Strain | Genotype | Reference |
|---|---|---|
| CC-5816 | *MTP* | This work |
| 3-3 | *MTP* | This work |
| 1-1 | *MTP* | This work |
| CC-5908 | *ac17; atg17::aphVIII; MTP* | This work; Lin et al., 2018 |
| CC-5909 | *ac17; atg17::aphVIII; MTM* | This work; Lin et al. 2018 |
| *pf23-1* | *pf23; ac17; atg17::aphVIII; MTP* | This work; Huang et al., 1979<br>This strain is an outcross of the *pf23-1* strain (CC-1383). |
| *pf23-2* | *pf23::aphVII; ac17; atg17::aphVIII; MTM* | This work |
| *pf23-3* | *pf23::aphVII; ac17; atg17::aphVIII; MTM* | This work |
| *pf23-4* | *pf23::aphVII; ac17; atg17::aphVIII; MTP* | This work |
